# Ultra-thermostable RNA nanoparticles for solubilizing and high-yield loading of paclitaxel for breast cancer therapy

**DOI:** 10.1038/s41467-020-14780-5

**Published:** 2020-02-20

**Authors:** Sijin Guo, Mario Vieweger, Kaiming Zhang, Hongran Yin, Hongzhi Wang, Xin Li, Shanshan Li, Shuiying Hu, Alex Sparreboom, B. Mark Evers, Yizhou Dong, Wah Chiu, Peixuan Guo

**Affiliations:** 1 Center for RNA Nanobiotechnology and Nanomedicine, The Ohio State University, Columbus, OH 43210 USA; 2 Division of Pharmaceutics and Pharmacology, College of Pharmacy, The Ohio State University, Columbus, OH 43210 USA; 3 James Comprehensive Cancer Center, The Ohio State University, Columbus, OH 43210 USA; 4 Dorothy M. Davis Heart and Lung Research Institute, The Ohio State University, Columbus, OH 43210 USA; 5 Department of Bioengineering and James H. Clark Center, Stanford University, Stanford, CA 94305 USA; 6 Markey Cancer Center, University of Kentucky, Lexington, KY 40506 USA; 7 Division of Cryo-EM and Bioimaging, SLAC National Accelerator Laboratory, Stanford University, Menlo Park, CA 94025 USA

**Keywords:** Nanoparticles, Drug development

## Abstract

Paclitaxel is widely used in cancer treatments, but poor water-solubility and toxicity raise serious concerns. Here we report an RNA four-way junction nanoparticle with ultra-thermodynamic stability to solubilize and load paclitaxel for targeted cancer therapy. Each RNA nanoparticle covalently loads twenty-four paclitaxel molecules as a prodrug. The RNA-paclitaxel complex is structurally rigid and stable, demonstrated by the sub-nanometer resolution imaging of cryo-EM. Using RNA nanoparticles as carriers increases the water-solubility of paclitaxel by 32,000-fold. Intravenous injections of RNA-paclitaxel nanoparticles with specific cancer-targeting ligand dramatically inhibit breast cancer growth, with nearly undetectable toxicity and immune responses in mice. No fatalities are observed at a paclitaxel dose equal to the reported LD_50_. The use of ultra-thermostable RNA nanoparticles to deliver chemical prodrugs addresses issues with RNA unfolding and nanoparticle dissociation after high-density drug loading. This finding provides a stable nano-platform for chemo-drug delivery as well as an efficient method to solubilize hydrophobic drugs.

## Abstract (web summary)

Although paclitaxel is widely used as a chemotherapy, it suffers from poor solubility and toxicity issues. Here, the authors develop thermostable RNA nanoparticles and report the RNA-paclitaxel complex to display improved stability, drug loading capacity and solubility for improved targeted cancer therapy and reduced immune responses.

## Introduction

Historically, chemotherapy plays a significant role in cancer treatment. Many chemotherapeutic drugs, including paclitaxel (PTX), have been used clinically in cancer patient care, but their toxicity and water-insolubility have raised serious concerns^1,2^. Effective therapeutic dosages of PTX cause potential organ damage, myelosuppression, immunotoxicity, hypersensitivity, and neurotoxicity^2,3^. These undesired effects have challenged cancer chemotherapy. Due to low water solubility and permeability, PTX was categorized as the lowest bioavailability Class IV drug according to the Biopharmaceutical Classification System (BCS). The first formulation of PTX for intravenous (i.v.) administration was a blend of Cremophor EL (castor oil derivative) and dehydrated ethanol at 1:1 (v/v) as a solvent^4^. However, subsequent studies revealed that castor oil and its derivatives are toxic and caused severe side effects such as non-linear plasma pharmacokinetics due to entrapment and delayed clearance in the liver^5,6^.

Functional nanomaterials constructed via molecular self-assembly show enormous potential in biotechnology and biomedicine applications^7–10^. Nanoparticle-based delivery of small chemotherapeutics via biochemical conjugation, complexation, or prodrug formulation has demonstrated to be a promising strategy for cancer therapy, due to their capacity of refining the poor physicochemical properties of chemo-drugs^11^. Over the years, the field of RNA nanotechnology has emerged rapidly^7,12,13^. RNA was widely used as a unique and biocompatible material for constructing nanoparticles via bottom-up self-assembly^14–19^. The versatility of RNA nanostructures, including composition, structure, and functionality, can be precisely controlled. Typically, RNA nanoparticles can be conveniently functionalized with tumor-targeting ligands to achieve selective targeting and specific delivery to tumor^14,20,21^. This advantage allows the maximum utilization of therapeutics after systemic administration, as well as minimizing side effects caused by non-specific accumulation in other organs. As a result, therapeutic modules such as siRNA and anti-miRNA were conjugated to RNA nanoparticles and exhibited efficient tumor inhibition in various cancer models^20,21^. In addition, recent studies show that the immune responses of RNA nanoparticles, based on the phi29 packaging RNA, are size, shape, and sequence-dependent^22^. Thus, RNA nanoparticles can be tuned not to trigger immunostimulation, or to induce strong immune responses that can be potentially used for cancer immunotherapy or as vaccine adjuvant^22–24^. Taking these advantages into consideration, small chemotherapeutic drugs utilizing RNA nanoparticles as a drug delivery platform could exhibit tumor-specific therapeutic effects and reduce the side effects and toxicity that is currently observed.

The concept of thermodynamic stability, defined as the free energy, Δ*G°*, required for complex formation, is important in RNA nanotechnology^12^. We previously discovered a thermodynamically stable phi29 pRNA three-way junction (3WJ) motif which has been used as a scaffold to construct multifunctional RNA nanoparticles^25^. This multivalent RNA can be modified with various chemical moieties^26^, which makes the covalent conjugation of chemo-drugs to RNA nanoparticles achievable^27^. However, increasing the drug loading capacity remains challenging for the current pRNA-3WJ nanoparticles. The limited drug loading capacity can be caused by steric hindrance that interferes with assembly, unfolding of RNA 2D or 3D structures after conjugation, or disassociation of RNA nanoparticles due to reduction of thermostability.

To address these concerns, we deliberately redesign our RNA vehicle to further elevate its thermostability and maximize the number of drugs that can be incorporated in the nanoparticle. An RNA four-way junction (4WJ-X) is constructed to achieve high-yield loading of drugs, as well as significantly solubilize the hydrophobic PTX for tumor inhibition. The engineered RNA 4WJ-X nanostructure is evolved from the pRNA-3WJ motif^25^ with much higher thermodynamic stability, displaying an extraordinary annealing temperature (*T*_a_) higher than 80 °C, far superior to pRNA-3WJ. Therefore, it completely addresses the concerns of reduced assembly efficiency as well as unfolding and dissociation over time after drug conjugation. The 4WJ-X nanostructures covalently conjugated with and without PTX are successfully formed with high structural stability and rigidity, as revealed by single-particle cryo-EM and 3D reconstruction. The resulting RNA-PTX complex retains its tertiary folding and independent functionalities both in vitro and in vivo, as demonstrated in the inhibition of triple negative breast cancer (TNBC) growth in animal trial. Currently, the major challenge in cancer therapy using PTX is not the anticancer drug efficacy, but its high toxicity. In this study, the RNA-PTX nanoparticles display undetectable toxicity or immune stimulation in mice. Our finding highlights the strong potential for using thermostable RNA-PTX nanoparticles as an efficient anticancer drug carrier.

## Results

### Construction of ultra-thermostable RNA nanostructure

**Fig. 1:**
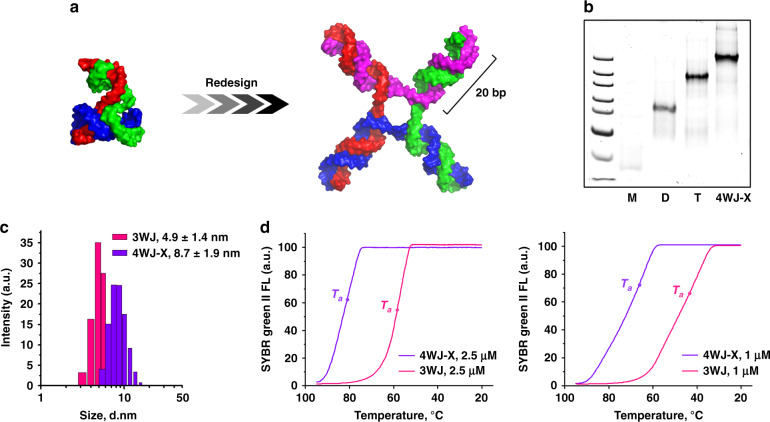
Design and construction of thermostable RNA 4WJ-X nanostructure. **a** Hypothetical 3D model of RNA 4WJ-X nanostructure (left), redesigned from pRNA-3WJ motif (right)^25,29^. **b** Step-wise self-assembly of 4WJ-X, evaluated by native polyacrylamide gel electrophoresis (PAGE) (size marker: ultra-low range DNA ladder; M, D, and T indicate RNA monomer, dimer, and trimer, respectively). **c** Size comparison of 4WJ-X (purple) and pRNA-3WJ (pink) by dynamic light scattering (DLS) (mean ± SD of one size distribution). **d** *T*_a_ comparison of 4WJ-X (purple) and pRNA-3WJ (pink) in representative annealing curves, measured by real-time-PCR (RT-PCR) (*n* = 3 independent samples over three independent measurements). Source data are provided as a Source Data file.

Attachment of chemicals or bioconjugation of foreign payloads to an RNA oligomer tends to induce steric constraints, and physical hindrance leading to annealing temperature (*T*_a_) alteration. Thus, using the previous RNA nanoparticles to carry sufficient chemo-drugs for cancer regression is challenging, due to reduced assembly efficacy and thermostability of the anticipated nanostructure after high-density loading with the drugs. As a means to improve RNA nanotechnologies’ capability in delivering high payloads of chemotherapeutic drugs, we intentionally evolved our nanostructure from pRNA-3WJ^25^ to 4WJ^28^ to maximize its thermodynamic stability as well as its drug loading capacity. The RNA nanoparticle used in the present work is a synthetic RNA 4WJ motif, with a hypothetical “X” shaped structure, composed of four RNA oligomers (Fig. 1a, b). It contains four 20 base-pair (bp) helices that are joined together at the junction domain. Each helix contains two functional domains: the core domain, and the payload domain. The nanoparticle core, the interior 8 bp of the helices, is designed to optimize the nanoparticle’s thermodynamic stability. The payload domain, the outer 12 bp of the helices, is designed to carry a high capacity of therapeutics with minimal effect on nanostructure stability. As a result, the RNA 4WJ-X displayed a larger hydrodynamic average diameter of 8.7 ± 1.9 nm, compared with 4.9 ± 1.4 nm for pRNA-3WJ (mean ± SD of one size distribution) (Fig. 1c). The *T*_a_ of 4WJ-X nanostructure (80.9 ± 2.7 °C at 2.5 μM, 66.1 ± 0.6 °C at 1 μM) was found to be 22 °C higher than that of pRNA-3WJ (58.4 ± 0.7 °C at 2.5 μM, 44.5 ± 1.6 °C at 1 μM) (*n* = 3 independent samples over three independent measurements, mean ± SD; representative annealing curves are shown in Fig. 1d). Next, 2′-propargyl modifications are incorporated at optimal intervals throughout the payload domain to introduce maximum alkyne moieties on the RNA oligomers for “click” coupling of azide (N_3_)-modified prodrugs without significant influence on assembly efficiency and thermostability. We then evaluated the capability of this ultra-thermodynamically stable RNA 4WJ-X nanostructure to carry high payloads of drug.

### RNA nanoparticles covalently loaded with high-density PTX

**Fig. 2:**
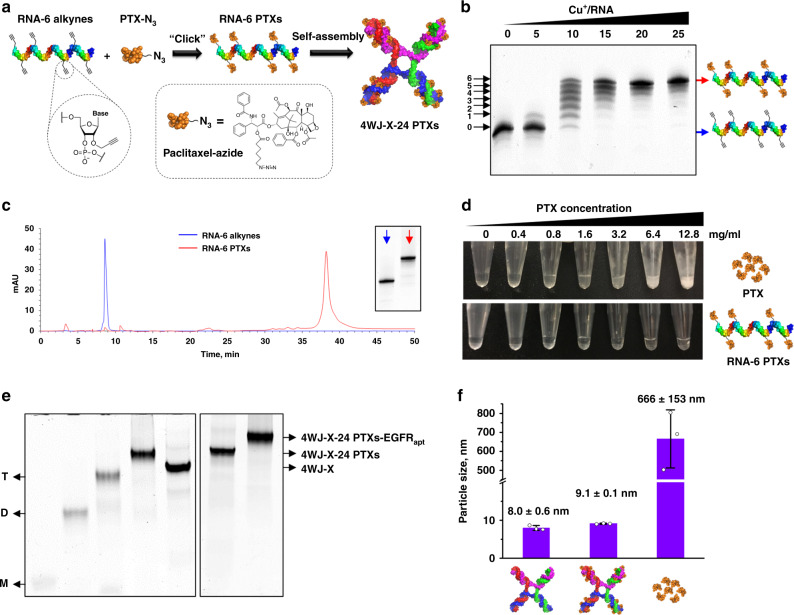
Conjugation of RNA-PTX and construction of 4WJ-X-24 PTXs nanoparticles. **a** Schematic of RNA-6 PTXs chemical conjugation and 4WJ-X-24 PTXs self-assembly. **b** Conjugating six PTXs to an RNA, evaluated by denaturing PAGE. **c** HPLC chromatogram (absorbance 260 nm) with an inserted gel image of RNA-6 alkynes (blue) and RNA-6 PTXs (red). **d** Turbidity changes of PTX and RNA-6 PTXs in aqueous solution at two-fold serial dilution. **e** Step-wise self-assembly of 4WJ-X-24 PTXs and 4WJ-X-24 PTXs-EGFR_apt_, evaluated by native PAGE (M, D, and T indicate RNA monomer, dimer, and trimer conjugated with PTX, respectively). **f** Size comparison of 4WJ-X, 4WJ-X-24 PTXs, and PTX in aqueous solution by DLS (*n* = 3 independent samples, mean ± SD). Source data are provided as a Source Data file.

Each RNA oligomer of the 4WJ-X was chemically synthesized with six alkyne groups throughout (RNA-6 alkynes). Thus, each RNA oligomer was site-specifically conjugated with six PTXs along the ribose-phosphate backbone (Fig. 2a). This design greatly improved the drug loading capacity of RNA, compared with previous RNA-drug mono-conjugates that could only introduce a single drug or chemical moiety at the 5′-end of RNA^15^. In addition, all RNA throughout the study were 2′-fluorine (2′-F) modified at pyrimidines to prevent enzymatic degradation in blood circulation and offer high chemical stability for in vivo applications. The successful PTXs multi-conjugation to RNA oligomer was evidenced by slower migration of RNA-multi-PTXs products in gel, due to the increased molecular size and hydrophobicity (Fig. 2b & Supplementary Fig. 1). Seven distinct bands were observed at a molar ratio of Cu^+^/RNA of 10, indicating that 0, 1, 2, 3, 4, 5, and 6 PTXs were conjugated to RNA, respectively (Fig. 2b). When this molar ratio was increased to 25, the maximum conjugation band (RNA-6 PTXs) dominated. The product was then purified and characterized by HPLC (Fig. 2c). Quantitative analysis of the HPLC spectrum revealed that the maximum conjugation efficacy for RNA-6 PTXs was 46.9% (Supplementary Fig. 2).

The unsatisfactory water-solubility of PTX has led to unfavorable pharmacokinetic/pharmacodynamic (PK/PD) profiles, and undesired side effects or toxicity^3,5,6^. RNA is a highly water-soluble biomaterial by nature. Dissolving RNA-6 PTXs conjugate in double-distilled water yielded clear solutions at increasing concentrations from 0.4 to 12.8 mg ml^−1^ (Fig. 2d). In contrast, solutions of free PTX drug showed increasing turbidity over the same concentration range. Therefore, the visibly clear solution of RNA-6 PTXs at 12.8 mg ml^−1^ of PTX suggests that the water-solubility of PTX is enhanced by at least 32,000-fold, compared with the reported water-solubility of PTX (<0.0004 mg ml^−1^)^30^. This significant enhancement demonstrates that RNA can greatly solve the water-insolubility problem of PTX and other chemo-drugs for administration in clinical trials.

When four single-stranded RNA-6 PTXs, each carrying six PTXs, were assembled into the 4WJ-X, the overall drug loading capacity was increased to 24 (4WJ-X-24 PTXs) (Fig. 2a). Step-wise self-assembly at equimolar oligo ratio resulted in clean gel bands with no visible free single-stranded RNA, demonstrating an extremely high assembly efficiency of the nanoparticles (Fig. 2e). DLS measurements of 4WJ-X-24 PTXs revealed an average hydrodynamic diameter of 9.1 ± 0.1 nm (*n* = 3 independent samples, mean ± SD) (Fig. 2f), slightly larger than the one without PTX, due to the addition of PTXs. More importantly, the low variation in the repeatable size measurements suggests a homogeneous assembly of the 4WJ-X-24 PTXs complex, indicating the RNA nanoparticles dramatically solubilized PTX in aqueous solution, compared with the large variation and heterogeneous size of free PTX in aqueous solution (Supplementary Fig. 3a-c). The zeta potential of the 4WJ-X-24 PTXs nanoparticles was −22.2 ± 4.64 mV (mean ± SD of the zeta potential distribution), indicating that the negatively charged property of RNA was well-retained after PTX conjugation (Supplementary Fig. 3d). In addition, PTX is covalently linked to RNA by an ester bond linkage (Fig. 2a), which can be cleaved by esterase hydrolysis. In vitro drug release assay showed that PTX were gradually released from the 3WJ-PTX nanoparticles after incubation in 50% fetal bovine serum (FBS) (Supplementary Fig. 4).

### Single-particle cryo-EM and 3D reconstruction

**Fig. 3:**
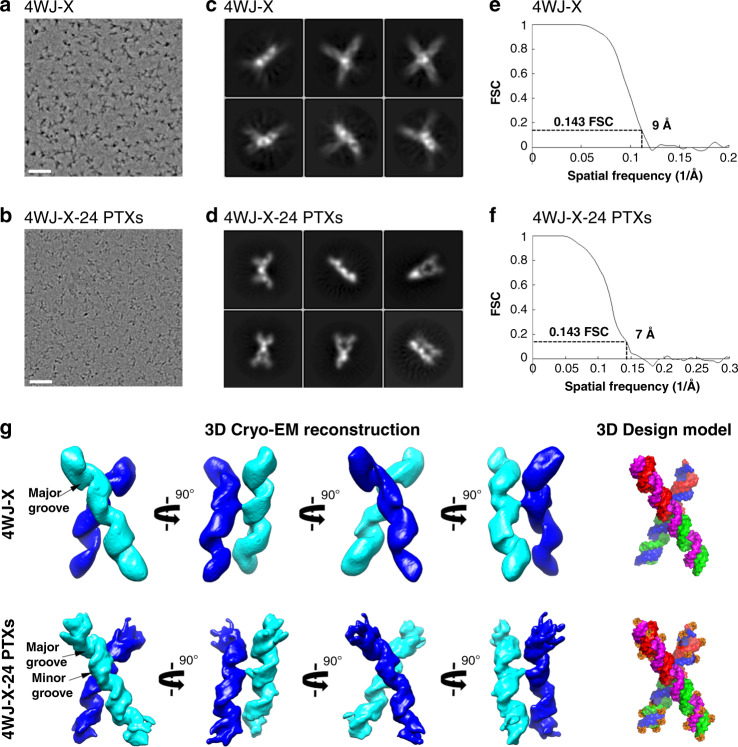
Comparison of 4WJ-X and 4WJ-X-24 PTXs nanoparticles by single-particle cryo-EM. **a** Representative motion-corrected cryo-EM micrographs of 4WJ-X and **b** 4WJ-X-24 PTXs (scale bar: 500 Å). **c** Reference-free 2D class averages of 4WJ-X and **d** 4WJ-X-24 PTXs computed in Relion. **e** Gold standard Fourier shell correlation (FSC) plots to measure resolution at FSC = 0.143 for the 3D reconstruction of 4WJ-X and **f** 4WJ-X-24 PTXs. **g** 3D reconstructed cryo-EM maps of 4WJ-X and 4WJ-24 PTXs in four views, with a Supplementary Movie 1. A 3D design model is shown. Source data are provided as a Source Data file.

As a rapidly growing technique in structural biology, cryo-EM single-particle analysis has been used for the validation of many DNA or RNA structures^31–34^. This technique was used to evaluate the structures of 4WJ-X and 4WJ-X-24 PTXs nanoparticles in vitrified solution. The cryo-EM images for both nanoparticles clearly revealed individual particles without any obvious aggregation (Fig. 3a, b). The 2D class averages showed different views of the 4WJ-X and 4WJ-X-24 PTXs objects with the clear major grooves RNA structural feature (Fig. 3c, d). After several rounds of the 2D/3D classification, over 100,000 and 120,000 selected particles for 4WJ-X and 4WJ-X-24 PTXs, respectively, were used for final 3D refinement without any symmetry applied. Compared with 4WJ-X, the 4WJ-X-24 PTXs complex displayed higher structural stability, resulting in the final map with resolution of 9 Å for 4WJ-X and of 7 Å for 4WJ-X-24 PTXs, respectively (Fig. 3e, f). The 3D reconstructions showed the “X” shape (Fig. 3g); the major grooves of the RNA in both of the maps were well resolved while the minor grooves can only be observed in the 7 Å map of 4WJ-X-24 PTXs. The length of 4WJ-X is slightly shorter than that of 4WJ-X-24 PTXs, which is consistent with the above DLS experiments and indicates that the PTXs contribute to the extended densities at the end of RNA duplexes (Supplementary Movie 1). Interestingly, the “X” shape is a two-layered structure with two RNA duplexes stacking on top of one another, connected by a short linker. The nucleotides at the junction area possibly function as the linker to build a “bridge” in 3D space (Supplementary Fig. 5). Taken together, these results confirmed the formation, stability, and rigidity of the RNA 4WJ-X nanoparticles with and without PTX at the sub-nanometer scale.

### RNA nanoparticles with high-density PTX remain thermostable

**Fig. 4:**
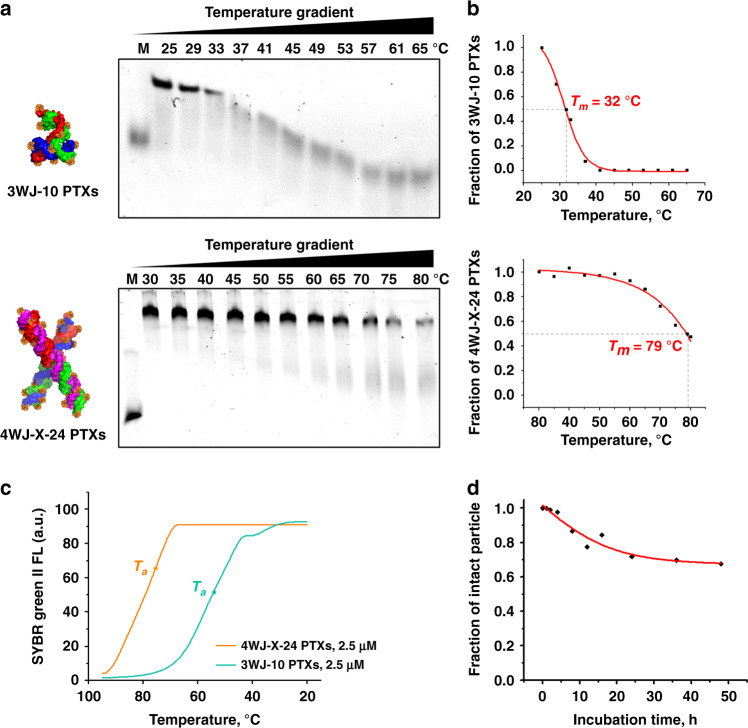
Thermodynamic and chemical stability of 4WJ-X-24 PTXs nanoparticles. **a** Representative TGGE showing the *T*_m_ of 3WJ-10 PTXs (M = 20 nucleotides monomer) and 4WJ-X-24 PTXs (M = 40 nucleotides monomer) nanoparticles. **b** Quantitative calculation of *T*_m_ from the representative TGGE for 3WJ-10 PTXs and 4WJ-X-24 PTXs nanoparticles. **c** *T*_a_ comparison of 4WJ-X-24 PTXs (orange) and 3WJ-10 PTXs (green) nanoparticles in a representative annealing curve, measured by RT-PCR (*n* = 3 independent samples over three independent measurements). **d** Enzymatic stability curve of 4WJ-X nanoparticles after incubation in 50% FBS at 37 °C over time points. Source data are provided as a Source Data file.

When nanoparticles are systemically administrated to the body, structural stability of nanoparticles is critical to reach favorable PK/PD profiles and reduce side effects. Thermodynamically stable RNA nanostructures have been reported^21,25^. However, conjugation of chemo-drugs onto RNA nanoparticles could interfere with base-pairing, affect RNA folding, block nanoparticle assembly, and decrease the melting temperature (*T*_m_). Typically, PTX displays a large molecular structure and contains more aromatic rings, compared with ribonucleotides (Fig. 2a). The molecular weight of a PTX-N_3_ is about three times larger than that of a ribonucleotide. Therefore, stronger thermodynamic stability of the RNA vehicle is required to overcome the physical and chemical hindrance caused by PTX conjugation. As revealed by temperature-gradient gel electrophoresis (TGGE), the 4WJ-X-24 PTXs nanoparticles displayed a very high *T*_m_ of 79 °C (Fig. 4a, b). However, after conjugation with 10 PTXs, the *T*_m_ of resulting 3WJ-10 PTXs nanoparticles rapidly decreased to 32 °C, which is lower than the physiological temperature. A similar trend was observed from nanoparticle annealing curve, the *T*_a_ of the 4WJ-X-24 PTXs nanoparticles was 75.1 ± 0.7 °C at 2.5 µM, far higher than the 53.2 ± 5.1 °C of 3WJ-10 PTXs (*n* = 3 independent samples over three independent measurements, mean ± SD; a representative annealing curve is shown in Fig. 4c). The difference between *T*_a_ and *T*_m_ was probably caused by the different mechanisms for RT-PCR and TGGE to monitor the annealing and melting process, respectively. Since the *T*_m_ of RNA will be influenced by Mg^2+^ concentration^35^, TGGE was run in a Mg^2+^-free condition to reveal the true *T*_m_ value. Consistent results were observed by comparing the nanoparticles in a denaturing urea gel assay. Both 4WJ-X and 4WJ-X-24 PTXs nanoparticles remained stable without dissociation in the presence of 8 M urea, a strong nucleic acid denaturing condition, but the 3WJ-10 PTXs significantly dissociated to its single-stranded RNA fragments (Supplementary Fig. 6). These results clearly demonstrate that RNA 4WJ-X exhibits ultra-high thermodynamic stability, thus can serve as a robust scaffold to conjugate and deliver multiple chemo-drugs for in vivo applications. Moreover, the RNA nanoparticles in the present study were chemically modified with 2′-fluoro, which offers significant resistance to enzymatic degradation (Supplementary Fig. 7). Incubation of the 4WJ-X nanoparticles in 50% FBS for 48 h resulted in 67.6% of intact nanoparticles and thus a projected half-life of more than 2 days in vivo (Fig. 4d).

### Significant inhibition of tumor by RNA-PTX nanoparticles

**Fig. 5:**
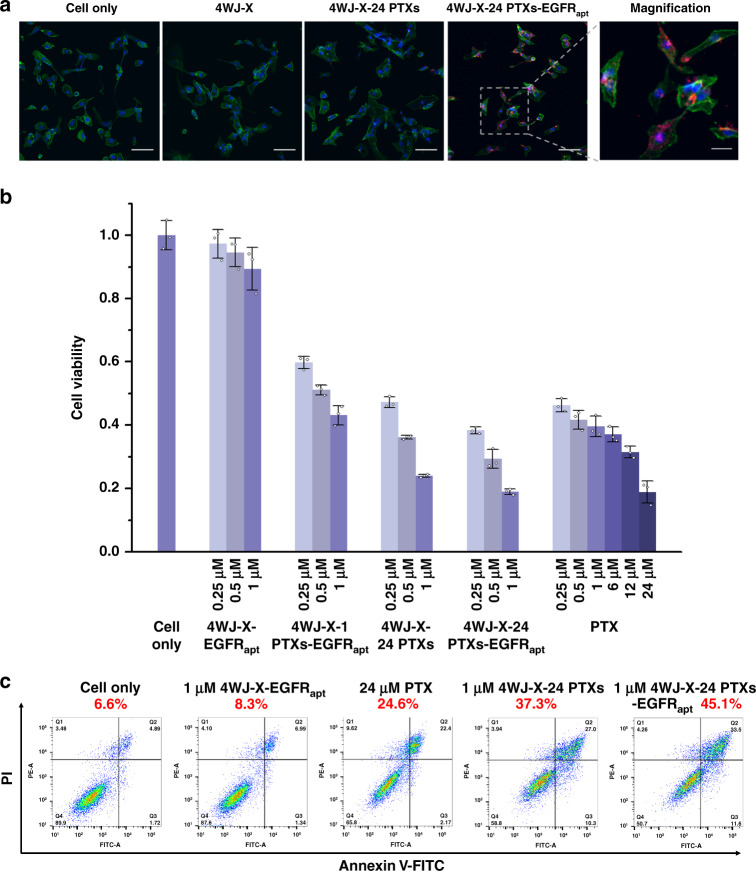
In vitro cell binding, cytotoxicity and apoptotic effects of 4WJ-X-24 PTXs nanoparticles. **a** In vitro cell binding of 4WJ-X-24 PTXs-EGFR_apt_ nanoparticles, shown by confocal microscopy (blue: nucleus; green: cytoskeleton; red: RNA nanoparticles. Scale bar: 100 μm for original images, and 20 μm for magnified image). **b** In vitro cytotoxicity study of 4WJ-X-24 PTXs-EGFR_apt_ nanoparticles by MTT assay (*n* = 3 independent samples, mean ± SD). **c** In vitro apoptosis effect of 4WJ-X-24 PTXs-EGFR_apt_ nanoparticles by propidium iodide (PI)/Annexin V-FITC dual staining and fluorescence-activated cell sorting (FACS) analysis (Q2 = Annexin V-FITC and PI positive, indicating cells in late apoptosis or already dead; Q3 = PI negative & Annexin V-FITC positive, indicating early apoptotic cells). Source data are provided as a Source Data file.

Specific tumor targeting is one of the most vital prerequisites and advantages of using nanoparticles to treat cancer. Studies have documented that the overexpression of Epidermal Growth Factor Receptor (EGFR) is associated with high cancer cell proliferation and high risk of recurrence in patients receiving treatments^36^. The anti-EGFR RNA aptamer (EGFR_apt_) has been reported to specifically bind EGFR on the surface of MDA-MB-231 breast cancer cells^20,21,37^. To equip our nanoparticles with tumor targeting capability, this anti-EGFR aptamer was incorporated on one of the 4WJ-X strands to construct multifunctional 4WJ-X-24 PTXs-EGFR_apt_ nanoparticles (Fig. 2e & Supplementary Fig. 8). The Alexa647 fluorophore, serving as an imaging reporter, was attached to another 4WJ-X strand (Supplementary Fig. 9). Confocal microscope imaging showed that 4WJ-X-24 PTXs-EGFR_apt_ nanoparticles exhibited enhanced cell binding to MDA-MB-231 cells in vitro, compared with the control groups (Fig. 5a). To evaluate the cytotoxic effect, the MTT assay was performed to determine cell viability after treatments. The 4WJ-X-24 PTXs-EGFR_apt_ nanoparticles significantly inhibited MDA-MB-231 cancer cell growth at or above 250 nM (Fig. 5b), while 4WJ-X-24 PTXs without EGFR_apt_ and 4WJ-X-1 PTXs-EGFR_apt_ with mono-conjugation exhibited weaker cytotoxicity, and 4WJ-X-EGFR_apt_ without PTXs hardly affected cell growth. The in vitro cell apoptosis assay revealed that 45.1% of the cells underwent apoptosis after 24 h treatment with 4WJ-X-24 PTXs-EGFR_apt_ nanoparticles, in comparison to 4WJ-X-24 PTXs (37.3%), 4WJ-X-EGFR_apt_ (8.3%), and free PTX (24.6%) (Fig. 5c & Supplementary Fig. 10). Since all the RNA-6 PTXs conjugates were purified by HPLC prior to assembly, the concern of free PTX contamination can be elegantly eliminated. Traditionally, RNA nanoparticles are negatively charged, making them have minimal interaction with negatively charged cell membranes. This nature limits their capacity of passing through cell membranes for intracellular drug delivery. However, by incorporating anti-EGFR aptamer, our results suggest that the 4WJ-X-24 PTXs-EGFR_apt_ nanoparticles can specifically bind to MDA-MB-231 breast cancer cells. Thus, it is very likely that more PTX were transported into cells and released intracellularly, leading to stronger in vitro drug efficacy.

**Fig. 6:**
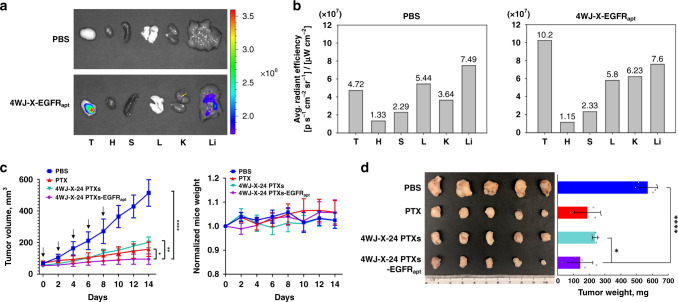
In vivo biodistribution and tumor inhibition of 4WJ-X-24 PTXs nanoparticles. **a** Representative organ images showing specific tumor targeting of Alexa Fluor 647 labeled 4WJ-X-EGFR_apt_ nanoparticles 8 h post-injection into mice bearing MDA-MB-231 xenograft (T: tumor, H: heart, S: spleen, L: lung, K: kidney, and Li: liver; Color scale: radiant efficiency, [p s^−1^ cm^−2^ sr^−1^] [μW cm^−2^]^−1^). Very low radiant mark was revealed in the liver of 4WJ-X-EGFR_apt_ treated sample. **b** Quantitative analysis of biodistribution in tumors and normal organs, quantified from the organ images. **c** Intravenous treatment of nude mice bearing orthotopic MDA-MB-231 xenografts with 4WJ-X-24 PTXs-EGFR_apt_ nanoparticles (purple) and control groups (turquoise: 4WJ-X-24 PTXs, red: PTX, blue: PBS) every other day for a total of five injections (8 mg kg^−1^, PTX per body weight, indicated by arrows). Mice body weight was monitored during the time course of treatments (*n* = 5 biologically independent animals, statistics was calculated by two-tailed unpaired *t*-test presented as mean ± SD, *p < 0.05, **p < 0.01, ****p < 0.0001; *p* = 0.038, 9.99 × 10^−4^, and 6 × 10^−6^ comparing 4WJ-X-24 PTXs-EGFR_apt_ to PTX, 4WJ-X-24 PTXs, and PBS, respectively). **d** Representative images of breast cancer tumors harvested from mice after treatments (*n* = 5 biologically independent animals, statistics was calculated by two-tailed unpaired *t*-test presented as mean ± SD, *p < 0.05, ****p < 0.0001; *p* = 0.033 and 2.2 × 10^−5^ comparing 4WJ-X-24 PTXs-EGFR_apt_ to 4WJ-X-24 PTXs, and PBS, respectively). Source data are provided as a Source Data file.

Next, in vivo tumor targeting and quantitative biodistribution of the EGFR_apt_-incorporated RNA nanoparticles were evaluated after systemic injection through the tail vein. Ex vivo images of tumor and healthy organs harvested from mice 8 h post-injection showed that the 4WJ-X-EGFR_apt_ nanoparticles strongly accumulated in tumor, with low and no accumulation in liver and other organs, respectively (Fig. 6a & Supplementary Fig. 11). Quantitative analysis of the organ images confirmed efficient tumor accumulation, compared with negative control (Fig. 6b). In vivo therapeutic effect of 4WJ-X-24 PTXs-EGFR_apt_ nanoparticles was evaluated using orthotopic TNBC xenograft tumors model. Mice treated with either 4WJ-X-24 PTXs nanoparticles, PTX (formulated in Cremophor EL/EtOH), or PBS served as control groups. Upon the treatment with 4WJ-X-24 PTXs at a dose of 8 mg kg^−1^ (PTX per mouse weight) every 2 days for a total of five dosages, the results showed a promising inhibitory effect on tumor growth in mice as monitored by tumor volumes, whereas control groups formed faster-growing tumors (Fig. 6c). The specific tumor inhibition was further validated from the tumors harvested from each group of 2-week post injections (Fig. 6d). In addition, the treatments were biocompatible and well tolerated in vivo, because no obvious toxicity was observed, indicated by the mice body weight over two-week post injections (Fig. 6c), and undetectable organ damage after systemic injections (Supplementary Fig. 12). Collectively, the data demonstrate that our thermostable RNA 4WJ-X-24 PTXs-EGFR_apt_ nanoparticles exhibited promising anti-tumor efficacy in vivo. The tumor inhibition of PTX loaded RNA nanoparticles was further confirmed using a KB cell xenograft tumor model (Supplementary Fig. 13). Folic acid (FA), a targeting ligand specifically interacts with the overexpressed folate receptor on KB cells, was incorporated at the same location as anti-EGFR aptamer on 4WJ-X nanoparticles. The result of intravenous treatment demonstrated effective tumor inhibition. The results confirmed the advantages of high thermodynamic stability and multivalent properties of RNA nanoparticles that enable the incorporation of different targeting ligands and therapeutics elements. All these results suggest that RNA nanoparticles as a powerful nano-delivery system for chemo-drugs displays great potential in cancer therapy.

### Undetectable immunostimulation and toxicity

**Fig. 7:**
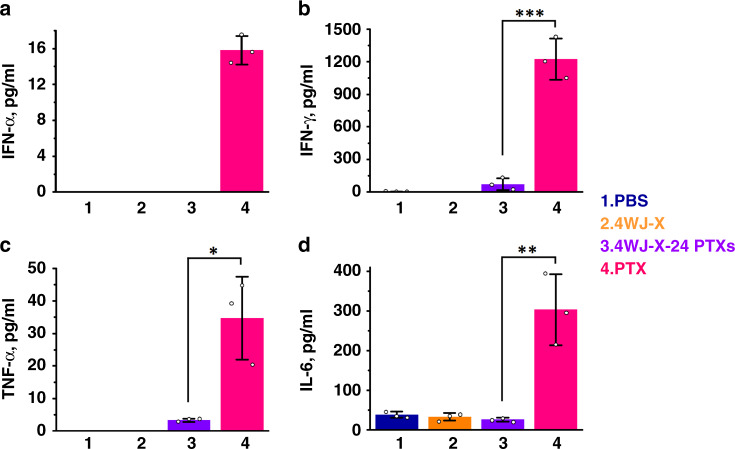
In vivo immunostimulation study of 4WJ-X-24 PTXs nanoparticles. Evaluation of **a** IFN-α, **b** IFN-γ, **c** TNF-α, and **d** IL-6 secretion in mice after systemic injection of 4WJ-X-24 PTXs nanoparticles, evaluated by enzyme-linked immunosorbent assay (ELISA) (*n* = 3 biologically independent animals, statistics was calculated by two-tailed unpaired t-test presented as mean ± SD, *p* = 5.4 × 10^−4^, 0.013, and 5.9 × 10^−3^ comparing 4WJ-X-24 PTXs to PTX for IFN-γ, TNF-α, and IL-6, respectively). Source data are provided as a Source Data file.

Toxicological and immunological evaluations are one of the most important drug evaluations prior to the use in the clinic^38^. Although PTX exhibits significant anti-tumor activity in vivo, many studies have indicated that the traditional formulation of PTX in polyethoxylated castor oil would cause severe non-specific toxicity, hypersensitivity, and other unwanted side effects in patients^5^. It was reported that these viscous solvents may adversely affect the pharmacokinetics of the drug due to entrapment in the liver and minimized drug clearance^6^. RNA nanoparticles, known as a safe and immunologically inert drug delivery carrier^22–24^, could be a great substitute for Cremophor EL. Here, in vivo production of pro-inflammatory cytokines, immunostimulation-related interferons (IFN), and chemokines were evaluated upon systemic administration of 4WJ-X-24 PTXs nanoparticles in mice. The results showed that i.v. injection of 4WJ-X-24 PTXs nanoparticles at the dose of 5 mg kg^−1^ induced undetectable or negligible tumor necrosis factor α (TNF-α) and interleukin 6 (IL-6) as well as type I IFN-α and type II IFN-γ production (Fig. 7). In contrast, PTX formulated in Cremophor EL/EtOH induced elevated production of these immune response indicators. Moreover, 25 types of chemokines, a group of pro-inflammatory mediators, were profiled upon the same treatments in vivo. Likewise, no significantly elevated production of most chemokines was observed for the 4WJ-X-24 PTXs nanoparticles (Supplementary Fig. 14). Several chemokines such as C10, complement C5/C5a, and MIP-1α showed elevated induction compared with the PBS group, indicating the nanoparticles might partially trigger some mediators’ secretion. The histological study indicates that no organ damage was caused by 4WJ-X-24 PTXs nanoparticles after systemic injection (Supplementary Fig. 12). These results suggest that 4WJ-X-24 PTXs nanoparticles exhibited good biocompatibility and no negative effects on important organs in vivo. Overall, it is concluded that 4WJ-X-24 PTXs nanoparticles did not trigger immune responses compared with the Cremophor EL formulated PTX, and did not induce toxicity to normal organs. Moreover, we compared the dose of 4WJ-24 PTXs nanoparticles with the LD_50_ (12 mg kg^−1^) of free PTX and no fatalities were found, confirming that RNA nanoparticles conjugated with PTX are safe and reduce side effects. The results were ascribed to the fact that RNA nanoparticles not only eradicate the need of toxic solvents as excipients and remarkably improve the water solubility, but also take full advantage of the favorable pharmacokinetic nature of the RNA nanoparticles.

## Discussion

An RNA 4WJ-X nanostructure with ultra-thermodynamic stability was constructed for high-yield loading of PTX for targeted cancer therapy. Each RNA nanoparticle loaded with 24 copies of covalently linked PTX was used as a prodrug, while remaining ultra-thermostability for in vivo applications. The water-solubility of PTX with RNA as the carrier was greatly enhanced in comparison to free PTX. Single-particle imaging by cryo-EM clearly showed the formation of “X” shaped RNA nanoparticles at the sub-nanometer scale. Animal trials in triple negative breast cancer model revealed that the RNA nanoparticles incorporated with anti-EGFR aptamer can specifically target tumor and significantly inhibit tumor growth after systemic injections. Currently, the hurdle in using PTX for cancer patient care is not the issue of PTX’s efficacy but its toxicity. The RNA-PTX nanoparticles here displayed extremely low or undetectable toxicity or immune responses in mice. This work demonstrates an effective method to address the insolubility of hydrophobic drugs in patient care. More importantly, it demonstrates a promising nano-platform for chemotherapeutic drug delivery by using thermostable RNA nanoparticles with the capabilities of high-yield drug payload, tumor-specific targeting, and reduced adverse effects for cancer therapy.

## Methods

### Synthesis and conjugation of RNA-PTX

PTX-N_3_ prodrug was synthesized by a reaction mixture of paclitaxel (Alfa Aesar), 6-azido-hexanoic acid (Chem-IMPEX), N,N′-dicyclohexyl-carbodiimide (Acros Organics), and 4-(dimethylamino) pyridine (Sigma-Aldrich) at 1:2:2:1 equivalent ratio in 20 mL dichloromethane as solvent. The reaction was run at room temperature with stirring for 36 h, followed by filtration and rotary evaporation to yield crude product. The crude product was purified by silica gel chromatography using n-Hexane:ethyl acetate as eluent. RNA-6 alkynes oligomers (one terminal 5′-alkyne and five internal 2′-propargyl) were synthesized via standard solid-phase RNA synthesis^26^ using 5′-hexynyl phosphoramidites (Glen Res. Corp.) and 2′-propargyl phosphoramidites (ChemGenes Corp.). RNA oligomers were purified by desalting using Glen Pak purification cartridges (Glen Res. Corp). The RNA sequences (lower-case letters indicate 2′-F nucleotides; letters labeled with * indicate 5′-hexynyl or 2′-propargyl nucleotides; underlined letters indicate anti-EGFR aptamer) are:

1. 3WJ a: 5′-uu*G ccA uGu* GuA uGu* GGG-3′
2. 3WJ b: 5′-c*cc AcA u*Ac uuu Gu*u GAu cc*-3′
3. 3WJ c: 5′-GGA u*cA Auc* AuG Gc*A A-3′
4. 4WJ-X a: 5′*-uuA GGu* AAA Gc*c Acc uGc AGG uGc uAc c*GA uGu* AAu uc*A A-3′
5. 4WJ-X b: 5′*-uuG AAu* uAc Au*c GGu AGc AcG GGc uGu Gc*G AGG c*uG AAc* AG-3′
6. 4WJ-X b-5′NH_2_: 5′NH_2_-C6-u*uG AAu* uAc Au*c GGu AGc AcG GGc uGu Gc*G AGG c*uG AAc* AG-3′
7. 4WJ-X c: 5′*-cuG uuc* AGc cu*c GcA cAG ccA GcA c*Gc Acc* uGA Au*A GG-3′
8. 4WJ-X c-EGFR_apt_: 5′*-cuG uuc* AGc cu*c GcA cAG ccA GcA c*Gc Acc* uGA Au*A GGu Gcc uuA GuA AcG uGc uuu GAu Guc GAu ucG AcA GGA GGc-3′
9. 4WJ-X d: 5′*-ccu Auu* cAG Gu*G cGu Gcu GGG cuG cAG Gu*G Gcu u*uA ccu* AA-3′

Conjugation of PTX to RNA was performed using copper(I)-catalyzed alkyne-azide cycloaddition (“Click chemistry”). RNA-6 alkynes resuspended in diethyl pyrocarbonate-treated water (DEPC-H_2_O) were thoroughly mixed with PTX-N_3_ that dissolved in 3:1 (v/v) dimethyl sulfoxide/tert-butanol, followed by adding freshly prepared “click solution” (a blend of copper(I) bromide/Tris[(1-benzyl-1H-1,2,3-triazol-4-yl)methyl]amine (Sigma-Aldrich) at a 1:2 molar ratio) at a final 1:15:20 molar ratio of RNA:PTX:Cu^+^. After reaction at room temperature overnight, the product was characterized by 16% (w/v) 8 M urea PAGE in TBE buffer (89 mM Tris base-borate, 2 mM EDTA) at 200 V for 1 h, and stained by ethidium bromide (EtBr, Sigma-Aldrich) and visualized on a Typhoon FLA 7000 (GE Healthcare). Gel images were analyzed by ImageQuant TL. The reaction was subsequently diluted with 1/10 volumes of 0.3 M sodium acetate and 2.5 volumes of 100% ethanol and incubated at −20 °C overnight for RNA precipitation. The precipitates were re-dissolved in DEPC-H_2_O and purified by Ion-Pair Reverse Phase HPLC in an Agilent PLRP-S 4.6 × 250 mm 300 Å column. PTX-labeled RNA was separated from unreacted RNA-6 alkynes in an H_2_O/Acetonitrile (ACN) ramp. HPLC data was collected by OpenLAB CDS. Fractions of RNA-6 PTXs were combined, dried and resuspended in DEPC-H_2_O for nanoparticle assembly.

### Construction of 4WJ-X-24 PTXs nanoparticles

The 3D computational model of 4WJ-X nanostructure was generated using Swiss PDB Viewer and PyMOL Molecular Graphics System. The 4WJ-X composed of four RNA strands is designed to lock into a four-strand assembly stabilized by base-pairing complementarity which generates four helix domains arranged around a nanoparticle core. 4WJ-X-24 PTXs nanoparticles were assembled by mixing four RNA-6 PTXs oligomers at equimolar concentrations in TES buffer (50 mM Tris pH = 8.0, 50 mM NaCl, 1 mM EDTA), followed by denaturing at 95 °C for 5 min and gradual cooling to 4 °C over the course of 1 h. Nanoparticle assembly was confirmed using a 12% (w/v) native PAGE in TBE buffer (89 mM Tris base, 200 mM boric acid and 2 mM EDTA) at 150 V for 1 h, and stained and visualized as described above. To construct 4WJ-X-24 PTXs-EGFR_apt_ nanoparticles, one of the RNA oligomers (4WJ-X c) was incorporated with an anti-EGFR aptamer^20,21,37^ at the 3′-end during synthesis.

### DLS measurement

The apparent hydrodynamic diameter and zeta potential of the RNA nanoparticles in 1 μM in TES buffer were determined using a Zetasizer Nano-ZS (Malvern Instrument) at 25 °C. The laser wavelength was 633 nm. Results were plotted with data points using Origin. For size comparison of 4WJ-X, 4WJ-X-24 PTXs, and PTX, three independent samples were measured and average particle size were obtained.

### *T*_a_ measurement by RT-PCR

Preassembled RNA nanoparticles were added to a 96-well plate with a final concentration of 2.5 μM and 1 μM. SYBR Green II dye (Invitrogen) was added to each well as a reporter dye for RNA nanoparticles formation at a final concentration of 20×. Samples were heated to 95 °C for 5 min as a denaturing process followed by a slow cooling ramp to 20 °C at a rate of 0.11 °C s^−1^ as an annealing process. Annealing curves of RNA nanoparticles were measured by Roche LightCycler^®^ 480 RT-PCR machine, and were plotted with data points using Origin. Three independent samples were measured over three independent experiments with similar results, and representative annealing curves are shown. *T*_a_ was determined by LightCycler 480 Software using the first derivative of the annealing profile.

### Cryo-electron microscopy data acquisition

Two samples of 4WJ-X nanoparticles, apo state (4WJ-X) and PTX-bound state (4WJ-X-24 PTXs), were diluted to a final concentration of ~20 µM. Three microliters of sample were applied onto glow-discharged 200-mesh R2/1 Quantifoil grids. The grids were blotted for 3 s and rapidly cooled in liquid ethane using Vitrobot Mark IV (ThermoFisher) at 22 °C and 100% humidity. The samples were screened using Talos Arctica cryo-electron microscope (ThermoFisher) operated at 200 kV and then imaged in a Titan Krios cryo-electron microscope (ThermoFisher) with GIF energy filter (Gatan) at a magnification of ×215,000 (corresponding to a calibrated sampling of 0.65 Å per pixel) for apo state and at a magnification of ×130,000 (corresponding to a calibrated sampling of 1.06 Å per pixel) for PTX-bound state using EPU (ThermoFisher). Micrographs were recorded with a Gatan K2 Summit direct electron detector, where each image is composed of 30 individual frames with an exposure time of 6 s and a specimen dose rate of 10 and 7.6 electrons per second per Å^2^ for apo and PTX-bound state, respectively. A total of 2780 movie stacks for apo state and 3800 movie stacks for the PTX-bound state were collected with a defocus range of 1.5–3.5 μm.

### Single-particle image processing and 3D reconstruction

All micrographs were motion-corrected using MotionCor2^39^ and CTF was determined using CTFFIND4^40^. All particles were autopicked using NeuralNet option in EMAN2^41^ and further checked manually, yielding 336,340 particles from selected 2618 micrographs for the apo state, and 406,408 particles from selected 3496 micrographs for the PTX-bound state. The particle coordinates were then imported to RELION^42^, where four rounds of 2D classification were performed to remove poor 2D class averages. The initial model was built using the “Ab initio 3D reconstruction” option in CryoSPARC^43^, and the 3D classification was performed in RELION. The final 3D refinement was performed in RELION using 106,775 particles for apo state and 125,909 particles for PTX-bound state, resulting in the corresponding maps with resolutions of 9 and 7 Å, respectively, based on the FSC of two independent particle data sets at a threshold of 0.143. Figures and movie were prepared using Chimera^44^.

### TGGE gel shift assay

Preassembled RNA nanoparticles were run in a 12% (w/v) native PAGE in TBE buffer at 100 V for 10 min at room temperature. TGGE (Biometra Gmbh) was subsequently performed by applying a gradient temperature perpendicular to the electrical current and run for 60 min at 20 W. The gel was stained and visualized as described above. Quantified values of bands for each nanoparticle were divided by the sum of the total values in corresponding lanes, calculated by ImageJ. Melting curves were plotted with quantified data points using Origin. *T*_m_ values were defined as the temperature at which 50% of the loaded nanoparticles dissociated.

### Enzymatic stability assay

Preassembled RNA nanoparticles were incubated at the final concentration of 1 μM in cell culture medium containing 50% (v/v) FBS at 37 °C for different time points through 48 h. The resulting samples were examined using a 3% agarose gel run at 100 V for 40 min and stained and visualized as described above. Quantification analysis was performed using ImageJ to calculate the percentage of intact nanoparticles (intensity of the band at a time point/intensity of the band at 0 h) for each time point. Enzymatic degradation curve was plotted with quantified data points using Origin.

### Cell culture

Human MDA-MB-231 cells were obtained from American Type Culture Collection (ATCC). Cells were grown and cultured in DMEM/F-12 medium (ThermoFisher Scientific) containing both 10% (v/v) FBS and 100 U ml^−1^ penicillin at 37 °C in humidified air environment containing 5% CO_2_.

### Confocal microscopy imaging

MDA-MB-231 cells were seeded on glass coverslips and cultured at 37 °C overnight. Alexa Fluor 647 labeled RNA nanoparticles were incubated with cells at a final concentration of 100 nM for 4 h at 37 °C. After washing twice with PBS buffer, cells were fixed with 4% formaldehyde and washed again, followed by treatment with 0.1% Triton X-100 (Sigma-Aldrich) in PBS buffer for 5 min and subsequent cytoskeleton staining with Alexa Fluor 488 phalloidin (ThermoFisher Scientific) for 30 min at room temperature. After rinsing with PBS buffer, the cells were mounted with ProLong^@^ Gold Antifade Reagent (Life Technologies Corp.) containing DAPI for cell nucleus staining and assayed on Olympus FV3000 confocal microscope (Olympus Corp.). Data were collected using Fluoview FV31S-SW.

### In vitro apoptosis assay

Cell apoptosis was studied using FITC Annexin V Apoptosis Detection Kit (BD Pharmingen) following manufacturer instructions. Briefly, 5 × 10^4^ MDA-MB-231 cells were seeded on a 24-well plate overnight. RNA nanoparticles and free PTX were added into cells at a final concentration of 1 μM. After 24 h incubation at 37 °C in a humidified 5% CO_2_ environment, cells were trypsinized to single cell suspension, washed with PBS buffer twice, and resuspended in 100 μL 1 × Annexin V-FITC binding buffer. Five microliters of Annexin V-FITC and 5 μL of PI were added into each sample and incubated at room temperature for 20 min. The samples were then added into flow tubes that contained 200 μL of 1 × binding buffer for FACS analysis by FACSCalibur™ flow cytometer within 1 h. Data were analyzed by FlowJo.

### In vitro cytotoxicity assay

Cell cytotoxicity was studied using CellTiter 96 Non-Radioactive Cell Proliferation Assay (Promega) following manufacturer instructions. Briefly, 5 × 10^3^ MDA-MB-231 cells were seeded on a 96-well plate overnight. RNA nanoparticles and free PTX were added into each well in triplicates at indicated concentrations. After incubation at 37 °C for 48 h in a humidified 5% CO_2_ environment. Fifteen microliters of MTT Dye Solution was added to each well and the plate was incubated at 37 °C in the dark for 4 h. Next, 50 μL Solubilization Solution/Stop Mix was added to each well for dissolving the crystal in the dark. The plate was incubated for 2 h at room temperature. Finally, the crystal in each well were fully dissolved to a uniformly colored solution and their absorbance at 570 nm was measured by Synergy 4 microplate reader (Bio-Tek). Data were collected by Gen5, and normalized data was plotted using Origin.

### Tumor xenograft animal model

All animal procedures were performed in accordance with the Subcommittee on Research Animal Care of The Ohio State University guidelines approved by the Institutional Review Board. Mice are housed in sterile environment, with all supplies (cages, bedding, feed, water bottles) autoclaved or irradiated. To generate the xenograft model, female athymic nu/nu mice, 4–8 weeks old, were purchased from Taconic BiosciencesFarm. TNBC tumor xenografts were established by injecting 2 × 10^6^ MDA-MB-231 cells/site resuspended in sterile PBS into the mammary fat pads of nude mice. When the tumor nodules had reached a volume of 75 mm^3^, the mice were used for tumor inhibition studies.

### In vivo biodistribution study

Alexa Fluor 647 labeled 4WJ-X-EGFR_apt_ nanoparticles (15 mg kg^−1^, RNA per body weight) were systemically administered via the tail vein into MDA-MB-231 tumor bearing mice. PBS-injected mice were used as fluorescence negative controls. The whole-body imaging of mice was conducted at 8 h using an IVIS system (XMRS) with excitation at 640 nm and emission at 680 nm. The mice were sacrificed at 8 h post-injection by the inhalation of CO_2_ followed by cervical dislocation, and major organs were collected and subjected to fluorescence imaging for the assessment of biodistribution profiles. The fluorescence imaging data of average radiant efficiency ([p s^−1^ cm^−2^ sr^−1^] [μW cm^−2^]^−1^) were quantitative by IVIS system (XMRS) program.

### In vivo tumor inhibition by 4WJ-X-24 PTXs nanoparticles

Mice with established tumor nodules were randomly divided into four groups (*n* = 5 biologically independent animals). Samples were administrated by i.v. injection in a total of 5 doses (8 mg kg^−1^, PTX per body weight) every other day. Tumor volume, calculated as (length × width^2^)/2, and mouse weight were monitored every other day. Curves were plotted with data points using GraphPad Prism. On day 14, the mice were sacrificed followed by tumor extraction. Tumors were weighted, and result was plotted with data points using Origin. Data were statistically analyzed by two-tailed unpaired *t*-test and presented as mean ± SD; *p < 0.05; **p < 0.01; ****p < 0.0001.

### In vivo cytokines induction evaluation

CD-1 mice (4–5 weeks old) were purchased from Charles River Laboratories. 4WJ-X-24 PTXs nanoparticles and control groups were administered into mice (*n* = 3 biologically independent animals) via i.v. injection at 5 mg kg^−1^ (PTX per body weight). Blood samples were harvested from mice 3 h post-injection by cardiac puncture and centrifugated at 12,800 × *g* for 10 min. Concentrations of cytokines in serum supernatant were examined in triplicates using Mouse ELISA MAX Deluxe sets (BioLegend) for TNF-α, IL-6, and IFN-γ, and using Mouse IFN-α ELISA Kit (R&D Systems) for IFN-α, following manufacturer provided protocols (1:200 dilution for all capture antibodies and detection antibodies). Results were plotted using Origin. Data were statistically analyzed by two-tailed unpaired *t*-test and presented as mean ± SD; *p < 0.05; **p < 0.01; ***p < 0.001.

### Statistics

Each experiment was repeated independently for at least three times for each sample tested, unless otherwise indicated. The results are presented as mean ± standard deviation (SD). Statistical differences were evaluated using two-tailed unpaired *t*-test with GraphPad software, and statistically significant differences are denoted as *p < 0.05, **p < 0.01, ***p < 0.01, and ****p < 0.0001. No adjustments were made for multiple comparisons.

### Reporting summary

Further information on research design is available in the Nature Research Reporting Summary linked to this article.

## Supplementary information

Supplementary Information

Description of Additional Supplementary File

Supplementary Movie 1

Reporting Summary

## Source Data

Source Data

## Data Availability

The source data underlying Figs. 1–7 and Supplementary Figs. 1–14 are provided in the source data file. Cryo-EM structures have been deposited to the Electron Microscopy Data Bank under accession codes EMD-20699 for 4WJ-X nanoparticles and EMD-20697 for 4WJ-X-24 PTXs nanoparticles, respectively. Other relevant data that support the findings of this study are available from the corresponding author upon reasonable request.
