# Supplementary material for: Ultra-thermostable RNA nanoparticles for solubilizing and high-yield loading of paclitaxel for breast cancer therapy
Source: Nat Commun. 2020 Feb 20;11:972. doi: 10.1038/s41467-020-14780-5 (PMC7033104; doi:10.1038/s41467-020-14780-5)

Supplementary Information

Ultra-thermostable RNA Nanoparticles for Solubilizing and High Yield Loading Paclitaxel for Breast Cancer Therapy

Guo et al.

List of Content:

A. Supplementary Methods

B. Supplementary Figures 1-14

C. Supplementary full scans of gels

A. Supplementary Methods

Alexa Fluor 647 labeling of RNA

RNA-6 alkynes-NH_2 oligomers (one terminal 5'-amine and six internal 2'-propargyl) were synthesized via standard solid-phase RNA synthesis using 5'-amino modifier C6-PDA (Glen Res. Corp.) and 2'-propargyl phosphoramidites (ChemGenes Corp.). After desalting purification, the RNA was mixed well with Alexa647-NHS Ester (ThermoFisher Scientific), dissolved in DMSO, at a molar ratio of 1:10, and added with 0.1 M bicarbonate (pH 8.2). The reaction was run at room temperature overnight, and the product (RNA-6 alkynes-Alexa647) was purified by HPLC to remove unreacted RNA and fluorophores.

Analysis of histological morphology

The mice in tumor inhibition study were sacrificed on day 14, and important organs including heart, liver, spleen, and kidney were excised for histological evaluation. Organs were frozen and embedded by medium at −20 °C, and were subsequently sectioned onto slices. The samples were then stained by hematoxylin and eosin (HE), and were observed by microscopy to evaluate potential pathological lesion in the organs.

Evaluation of *in vivo* chemokines induction

Concentrations of chemokines in serum supernatant, collected from mice as described in cytokines induction evaluation, were examined in duplicates using Mouse Chemokine Array Kit (R&D Systems), following the manufacturer's instruction. LiCor was used for blotting spot quantification. Results were plotted using OriginLab.

Subcutaneous KB tumor xenograft model and tumor inhibition

All animal procedures were housed and performed in accordance with the Subcommittee on Research Animal Care of The Ohio State University guidelines approved by the Institutional Review Board. To generate the

xenograft model, female athymic nu/nu mice, 4-8 weeks old, were purchased from Taconic Farm. Subcutaneous KB tumor xenografts were established by injecting 2×10^6 KB cells/site resuspended in sterile PBS into the left shoulder of nude mice. Mice were randomly divided into four groups (n=5 biologically independent animals). When the tumor nodules reached a volume of 25 mm^3, the mice were used for tumor inhibition studies. Samples were administrated by i.v. injection in a total of 3 doses (10 mg kg^{-1}, PTX per body weight) every other day. Tumor volume, calculated as (length × $width^2$)/2, were monitored every other day for a total 12 days. Data were statistically analyzed by two-tailed unpaired t-test and presented as mean ± SD; $**p < 0.01$; $****p < 0.0001$.

B. Supplementary Figures

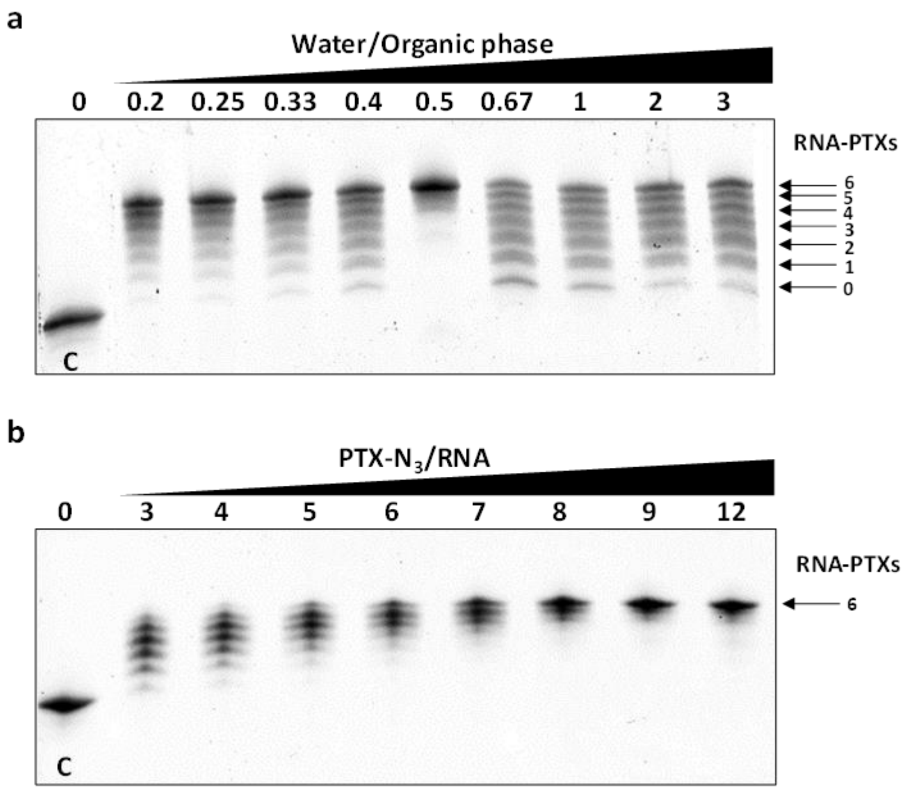

Supplementary Figure 1. Optimization of chemical conditions for RNA-PTXs conjugation. a. Conjugating PTX to RNA at different volume ratios of water/organic phase and **b.** molar ratios of PTX-N_3/RNA (C = RNA-6 alkynes control). Source data are provided as a Source Data file.

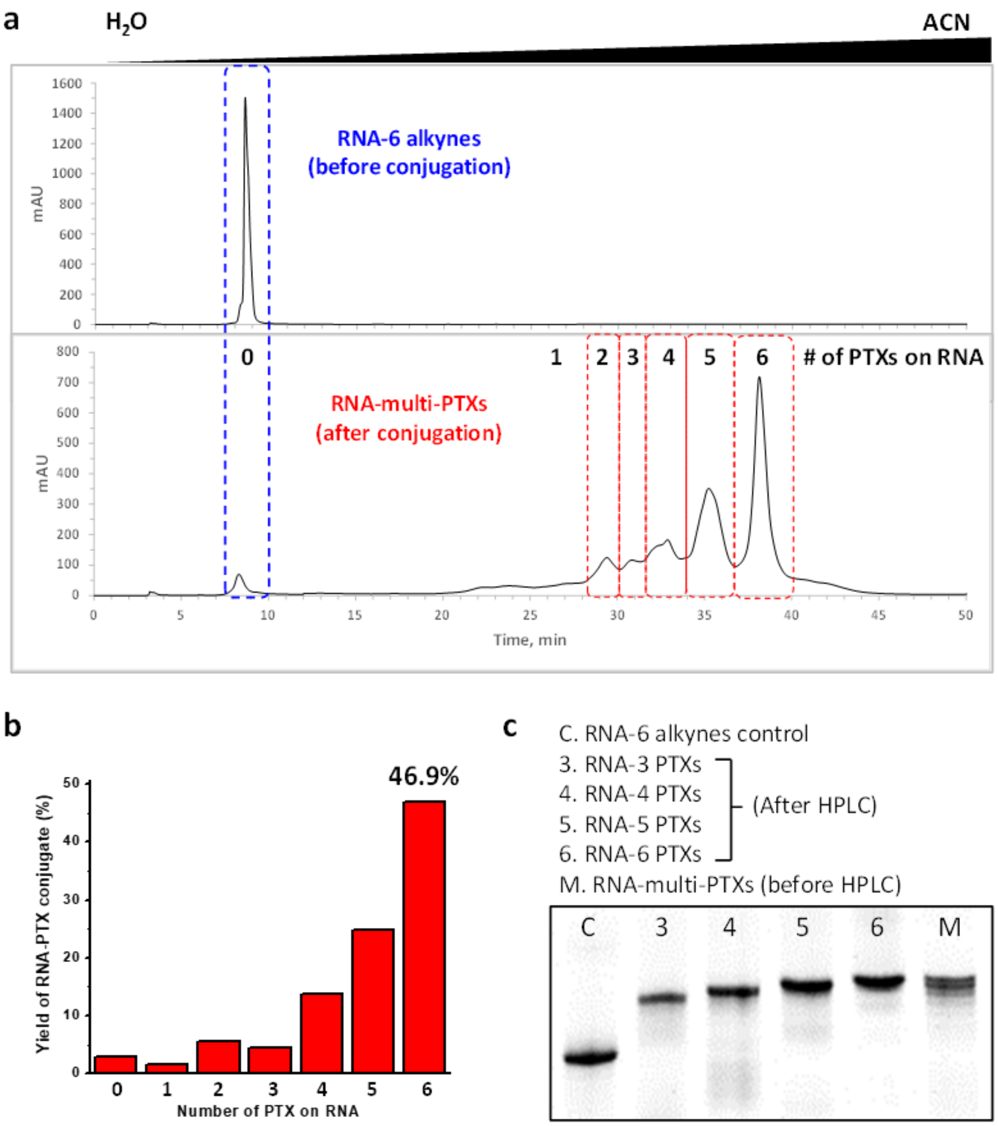

Supplementary Figure 2. Purification and quantification of RNA-PTXs conjugation. a. HPLC spectrum (absorbance 260 nm) of a representative RNA-multi-PTXs conjugation (blue: RNA-6 alkynes, red: RNA conjugated with different numbers of PTX). **b.** Conjugation efficiency of different numbers of PTXs to RNA, quantitatively analyzed from the HPLC spectrum in A. **c.** Purified RNA with different number of PTXs, evaluated by denaturing PAGE. Source data are provided as a Source Data file.

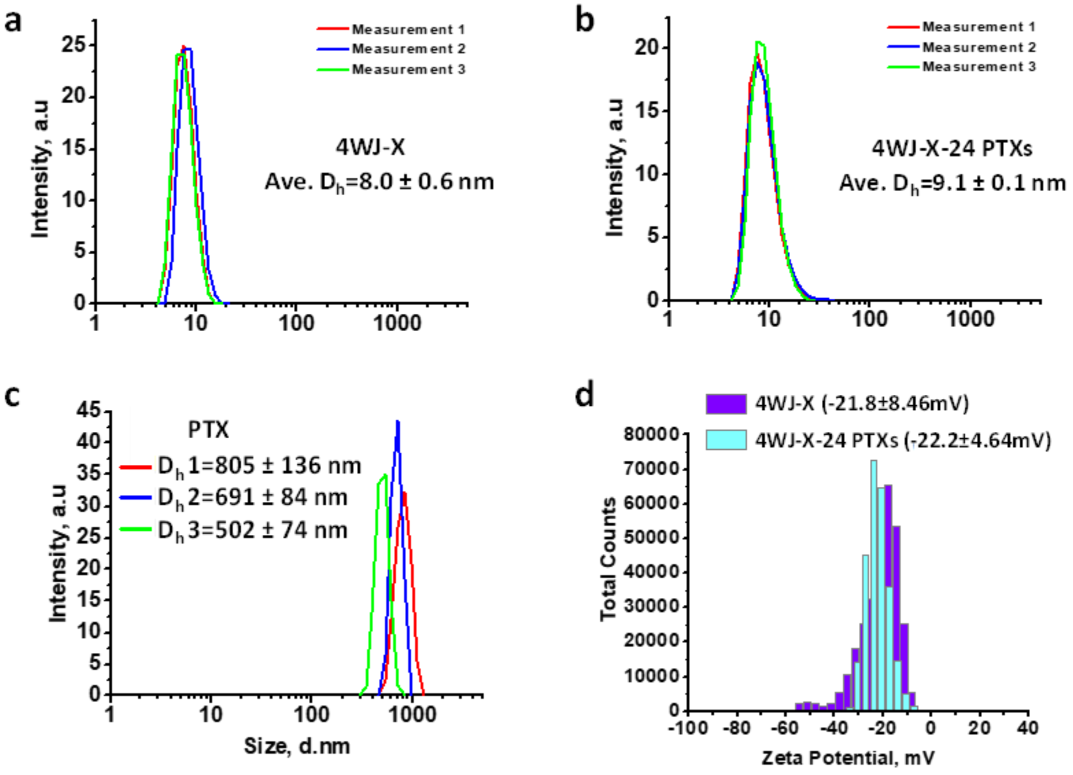

Supplementary Figure 3. Size distribution and zeta potential of RNA nanoparticles. a. Size distribution of 4WJ-X, **b.** 4WJ-X-24 PTXs, and **c.** PTX in aqueous solution, measured by DLS (n=3 independent samples, mean ± SD, D_h indicates hydrodynamic diameter). **d.** Zeta potential of 4WJ-X (purple) and 4WJ-X-24 PTXs (cyan) (mean ± SD of one zeta potential distribution). Source data are provided as a Source Data file.

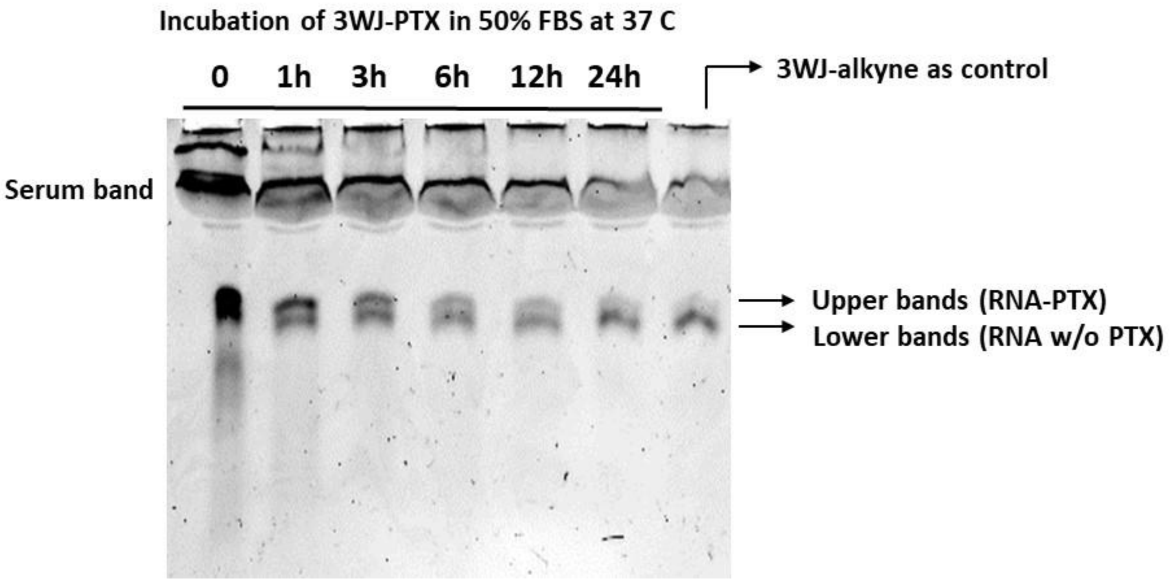

Supplementary Figure 4. ***In vitro* drug release of RNA nanoparticles.** 3WJ-PTX nanoparticles were incubated in 50% FBS at 37°C for different time points, and evaluated by native PAGE. Upper bands indicate intact RNA-PTX nanoparticles, while emerging lower bands indicate RNA nanoparticles after PTX release. Source data are provided as a Source Data file.

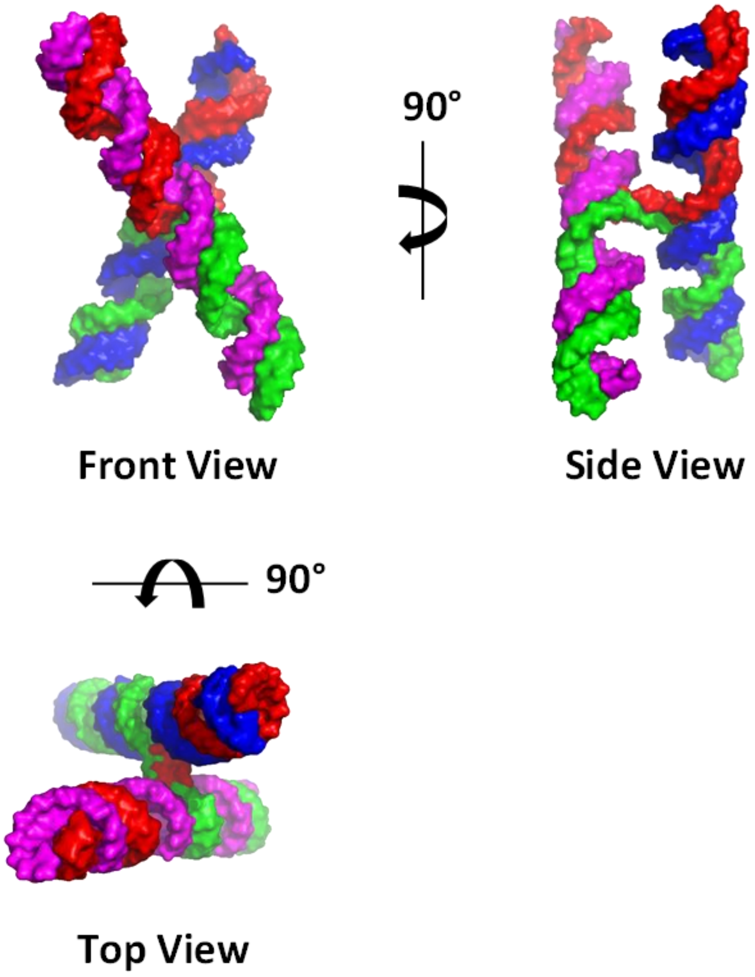

Supplementary Figure 5. 3D model of RNA 4WJ-X nanostructure. The 3D model of 4WJ-X nanostructure was constructed based on 3D cryo-EM reconstruction, and presented in three views.

	1	2	3	4	5	6	7	8
3WJ ssRNA	+							
3WJ		+						
3WJ ssRNA-4 PTXs			+					
3WJ-10 PTXs				+				
4WJ-X ssRNA					+			
4WJ-X						+		
4WJ-X ssRNA-6 PTXs							+	
4WJ-X-24 PTXs								+

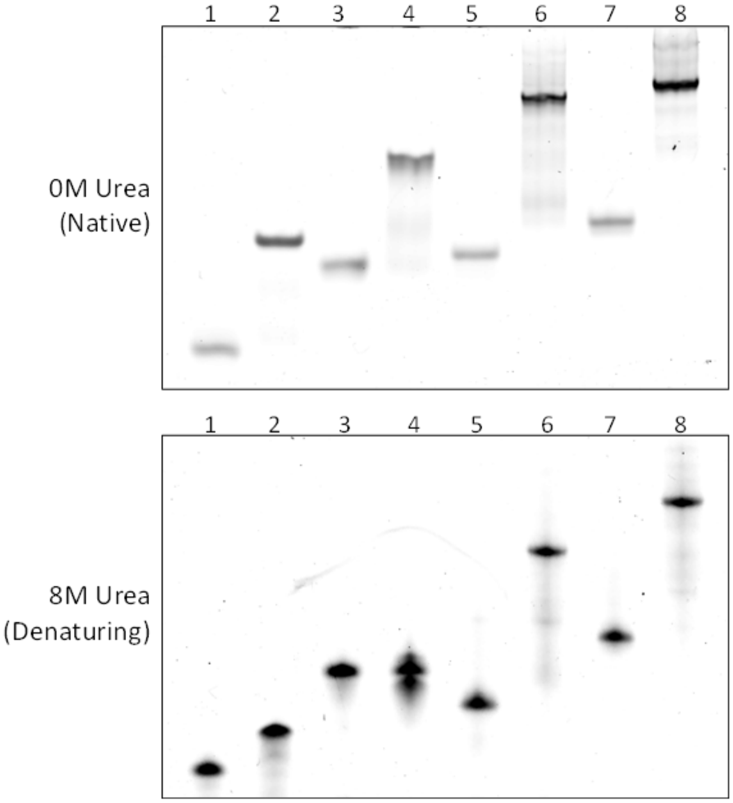

Supplementary Figure 6. Comparison of thermodynamic stability of RNA Nanoparticles. 3WJ, 3WJ-10 PTXs, 4WJ-X, and 4WJ-X-24 PTXs nanoparticles were examined in native and denaturing gel, respectively (ssRNA: single-stranded RNA). Source data are provided as a Source Data file.

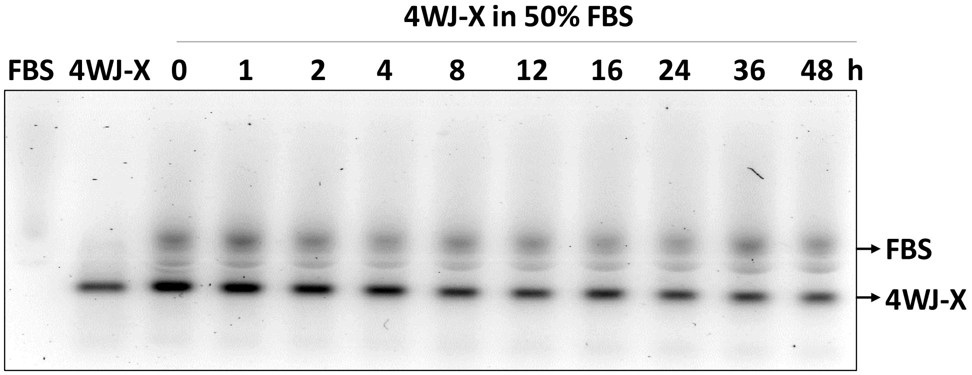

Supplementary Figure 7. Enzymatic stability study of 4WJ-X nanoparticles. A representative gel assay showing the enzymatic stability of 4WJ-X nanoparticles after incubation in 50% FBS at 37 °C over time points. Source data are provided as a Source Data file.

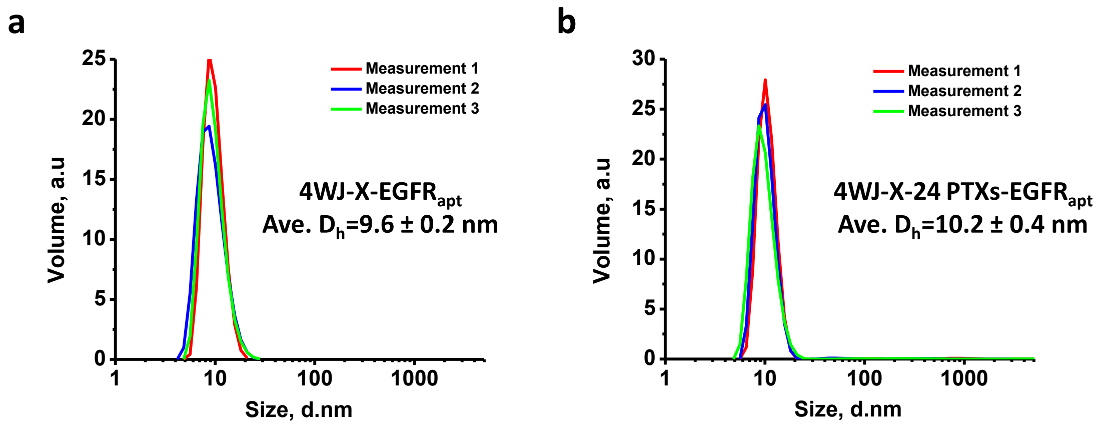

Supplementary Figure 8. Size distribution of RNA nanoparticles incorporated with EGFR aptamer. a. Size distribution of 4WJ-X-$EGFR_{apt}$ and **b.** 4WJ-X-24 PTXs-$EGFR_{apt}$, measured by DLS (n=3 independent samples, mean ± SD, D_h indicates hydrodynamic diameter). Source data are provided as a Source Data file.

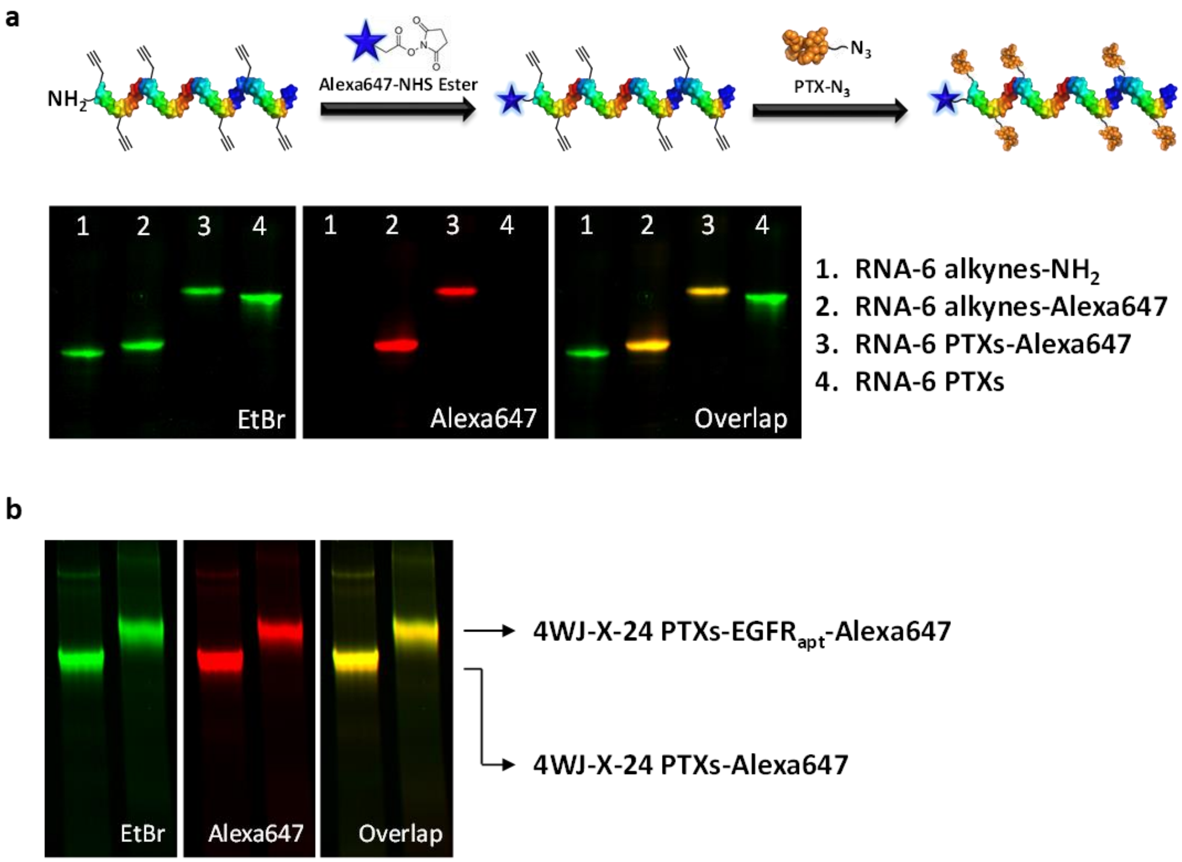

Supplementary Figure 9. Alex647 fluorophore labeling of RNA nanoparticles. **a.** Conjugation of Alexa647 fluorophore and PTX to RNA-6 alkynes-NH_2, evaluated by denaturing PAGE with different scanning wavelengths (green: EtBr, red: Alexa647, yellow: overlap). **b.** Self-assembly of 4WJ-X-24 PTXs-$EGFR_{apt}$-Alexa647 and 4WJ-X-24 PTXs-Alexa647 nanoparticles, evaluated by native PAGE with different scanning wavelengths. Source data are provided as a Source Data file.

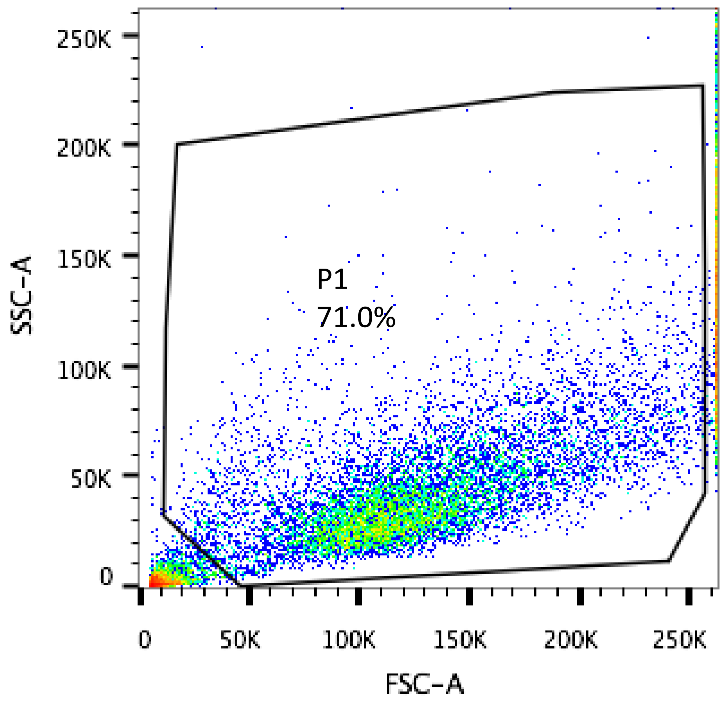

Supplementary Figure 10. Gating strategy to identify MDA-MB-231 cells with PI/Annexin V-FITC dual positive staining in cell apoptosis assay. Examples of gating cells (P1) for flow cytometry analysis study by size with forward scatter (FSC) and side scatter (SSC). Data is analyzed by FlowJo v7.6.2.

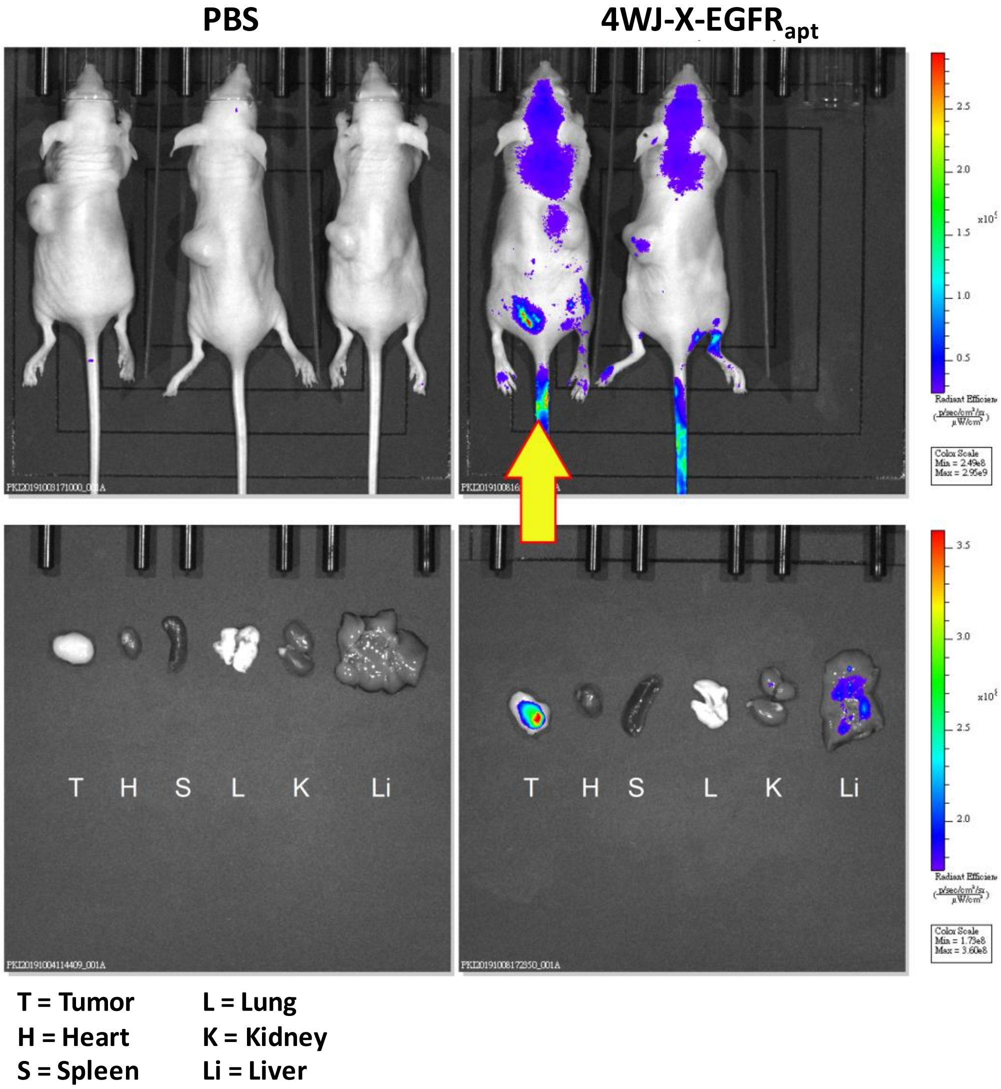

Supplementary Figure 11. ***In vivo* biodistribution of 4WJ-X-EGFR$_{apt}$ nanoparticles**. Whole-body and organ images showing specific tumor targeting 8 h after systemic injection of Alexa Fluor 647 labeled 4WJ-X-EGFR$_{apt}$ nanoparticles into mice with TNBC xenograft. Source data are provided as a Source Data file.

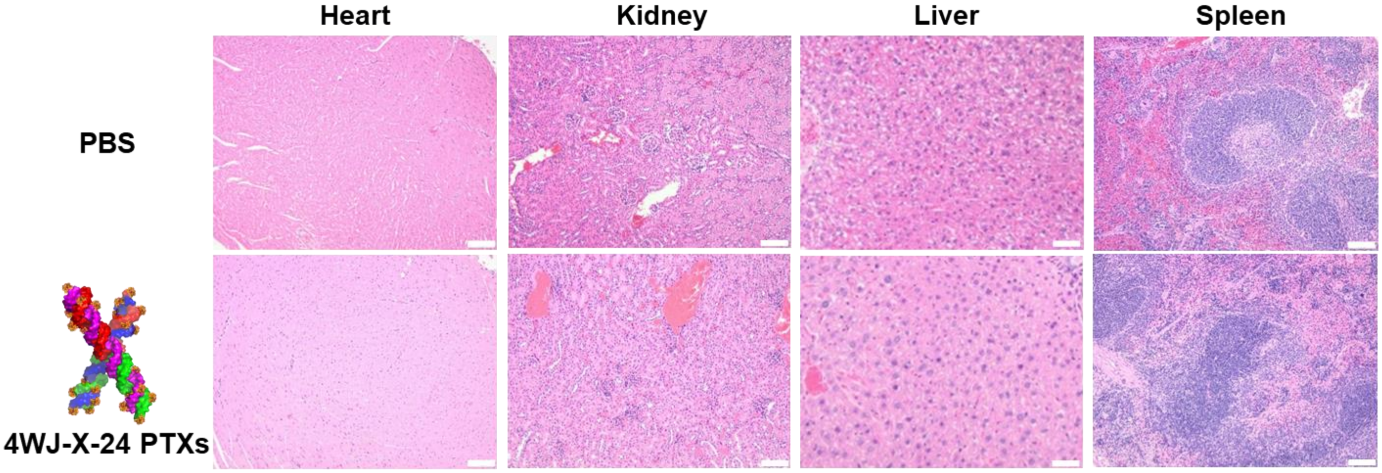

Supplementary Figure 12. Histological evaluation of normal organs after treatment with 4WJ-X-24 PTXs nanoparticles. After treatment, normal organs were excised from mice and evaluated by H&E staining (scale bar: 100 μm for heart, kidney, spleen, and 50 μm for liver). Source data are provided as a Source Data file.

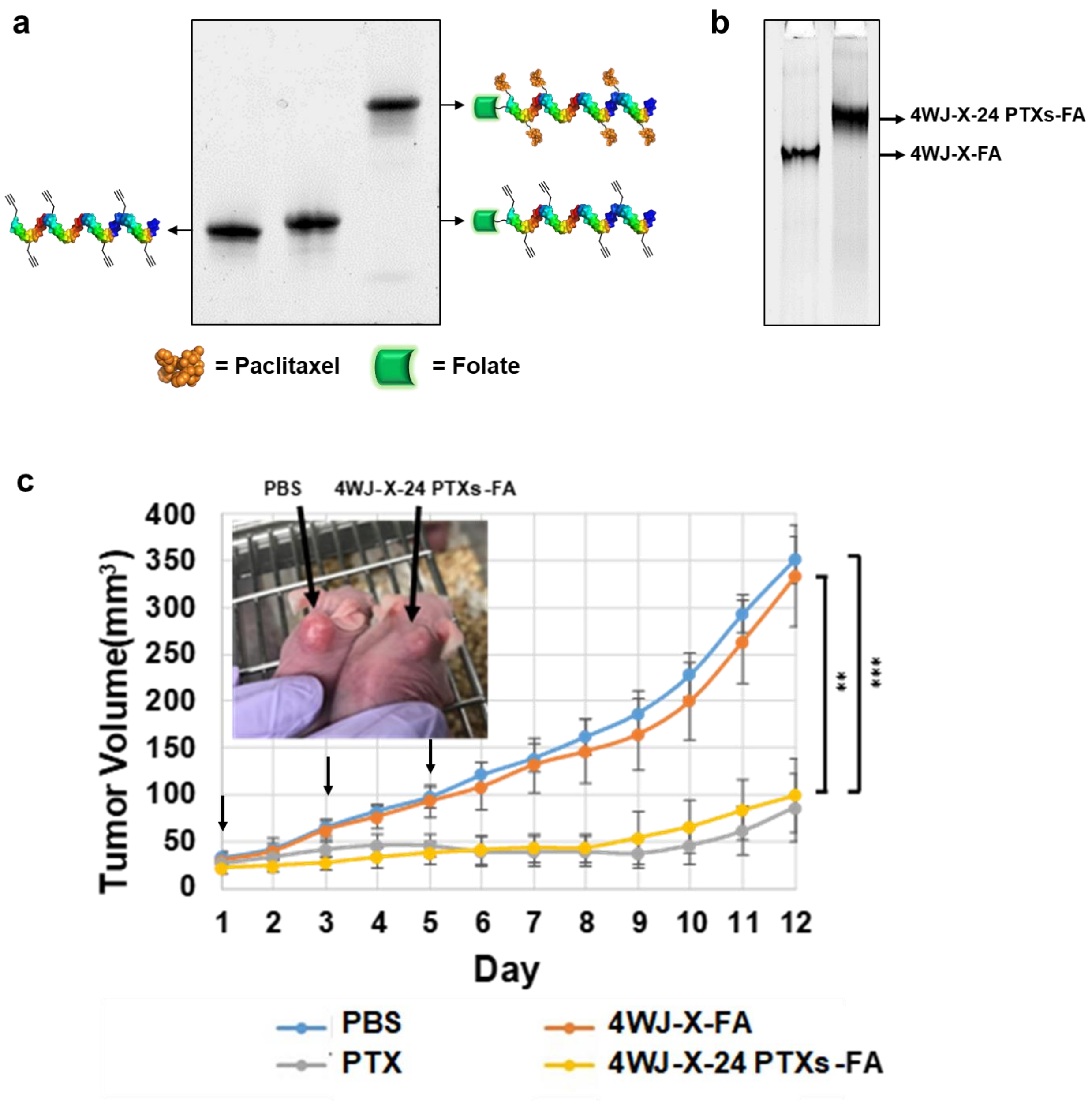

Supplementary Figure 13. Construction of 4WJ-X-24 PTXs-FA nanoparticles and its tumor inhibition on KB xenograft. a. Synthesis of RNA-6 PTXs-FA, evaluated by denaturing gel. **b.** Self-assembly of 4WJ-X-FA and 4WJ-X-24 PTXs-FA. **c.** Intravenous treatment of nude mice bearing KB subcutaneous xenografts with 4WJ-X-24 PTXs-FA nanoparticles (yellow) and control groups (orange: 4WJ-X-FA, gray: PTX, blue: PBS) every other day for a total three injections (10 mg kg^{-1}, PTX per body weight, indicated by arrows; n=5 biologically independent animals, statistics was calculated by two-tailed unpaired t-test presented as mean ± SD, $^{**}p < 0.01$, $^{***}p < 0.001$; p=0.0038, and 7×10^{-4} comparing 4WJ-X-24 PTXs-FA to 4WJ-X-FA, and PBS, respectively). Source data are provided as a Source Data file.

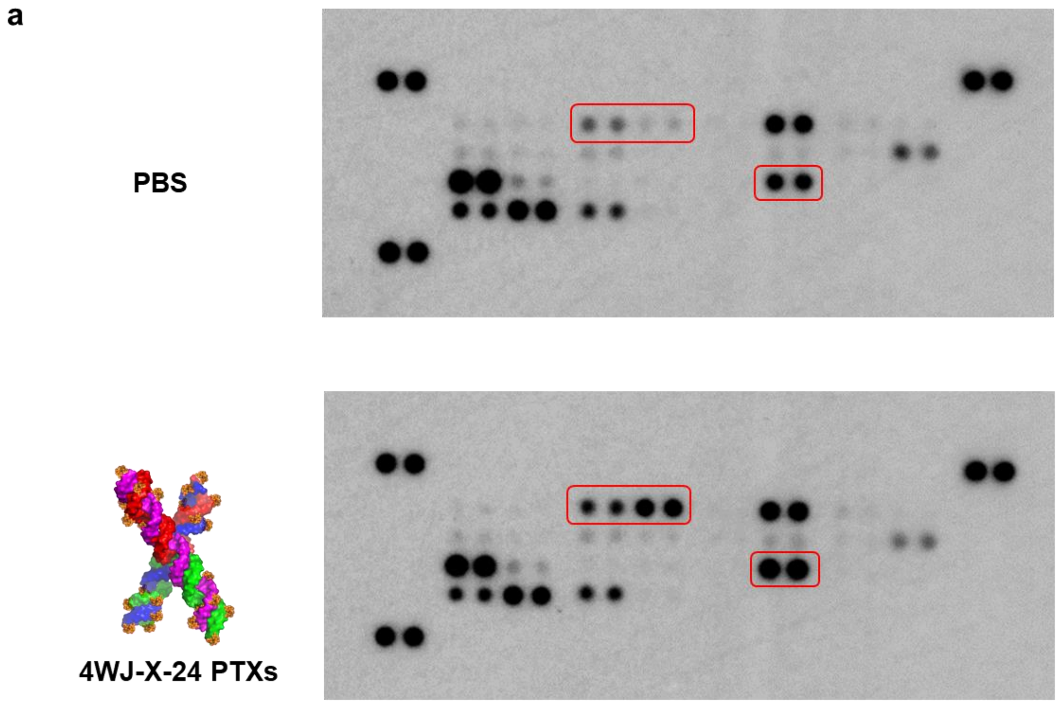

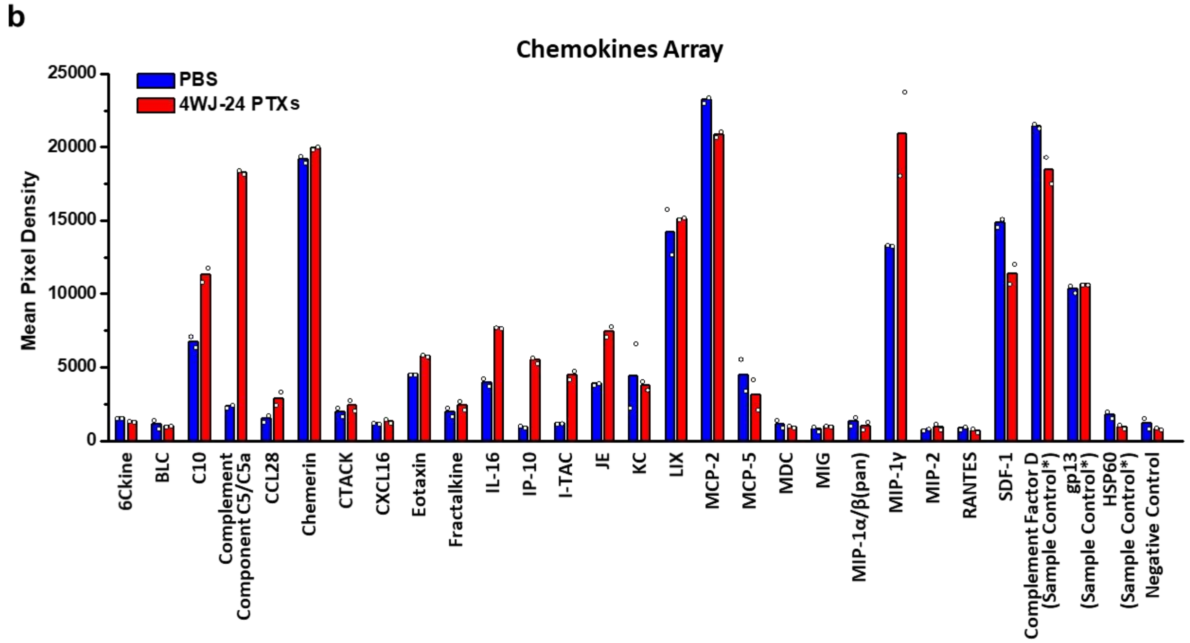

Supplementary Figure 14. *In vivo* chemokines induction study of 4WJ-X-24 PTXs nanoparticles. **a.** Blot image of *in vivo* chemokines profiling after treatments with 4WJ-X-24 PTXs (C10, complement C5/C5a, and MIP-1α are highlighted in red box with each sample duplicated from left to right, respectively) **b.** Quantitative analysis of the chemokines induction blot (n=2 independent samples, mean ± SD; red: 4WJ-X-24 PTXs, blue: PBS). Source data are provided as a Source Data file.

C. Supplementary full scans of gels

Figure 1b

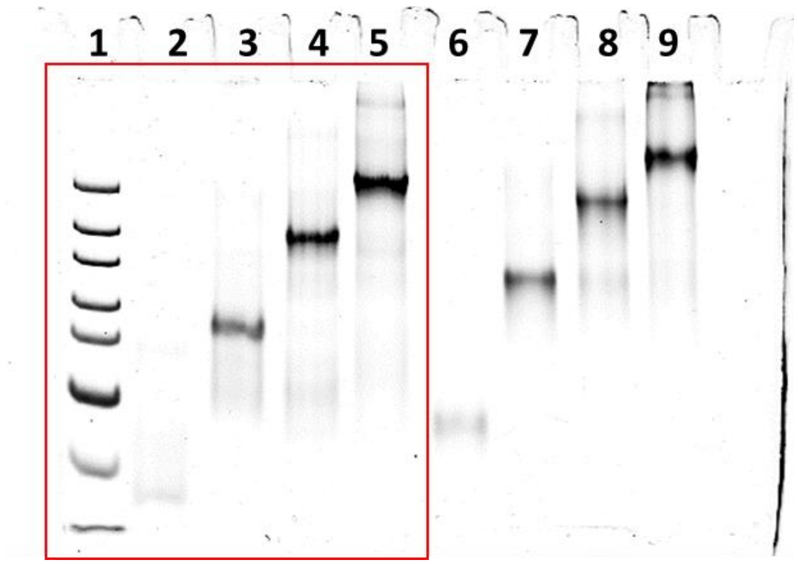

1. ultra-low range DNA ladder
2. 4WJ A
3. 4WJ A+B
4. 4WJ A+B+C
5. 4WJ A+B+C+D (4WJ-X)
6. 4WJ A-6 PTXs
7. 4WJ A+B-12 PTXs
8. 4WJ A+B+C-18 PTXs
9. 4WJ A+B+C+D-24 PTXs (4WJ-X-24 PTXs)

Figure 2b

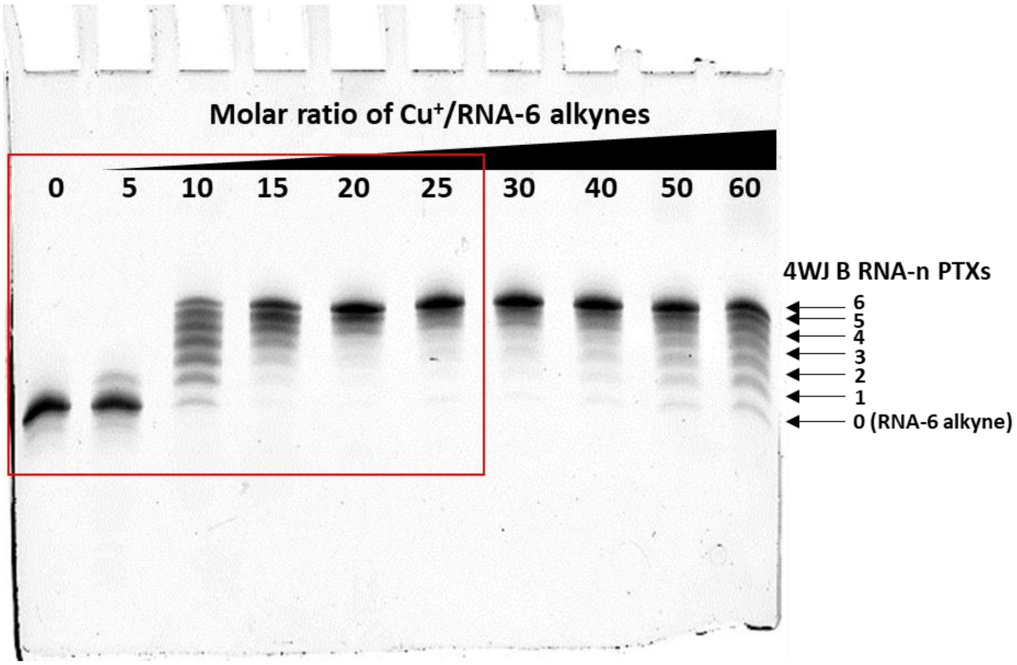

Molar ratio of Cu+/RNA-6 alkynes
0
5
10
15
20
25
30
40
50
60
4WJ B RNA-n PTXs
6
5
4
3
2
1
0 (RNA-6 alkyne)

Figure 2c (inset)

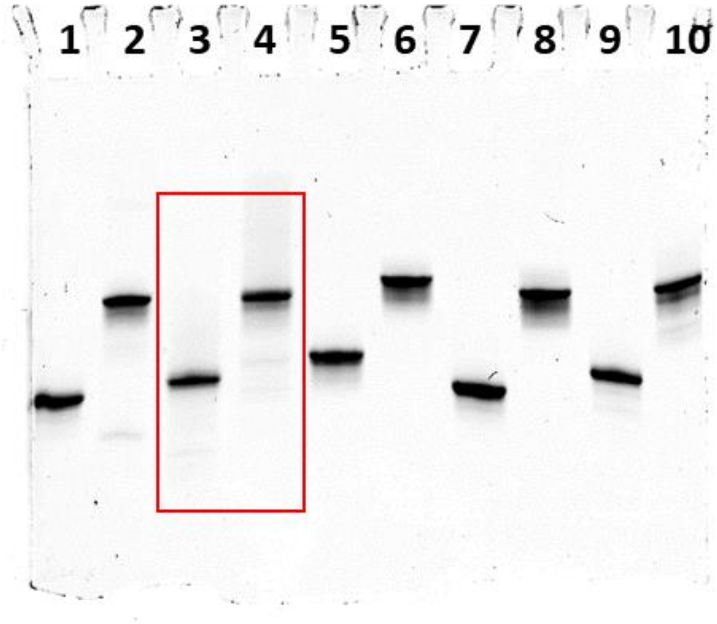

1. Extended 3WJ A-6 alkynes
2. Extended 3WJ A-6 PTXs
3. 4WJ A-6 alkynes
4. 4WJ A-6 PTXs
5. 4WJ B-6 alkynes
6. 4WJ B-6 PTXs
7. 4WJ C-6 alkynes
8. 4WJ C-6 PTXs
9. 4WJ D-6 alkynes
10. 4WJ D-6 PTXs

Figure 2e

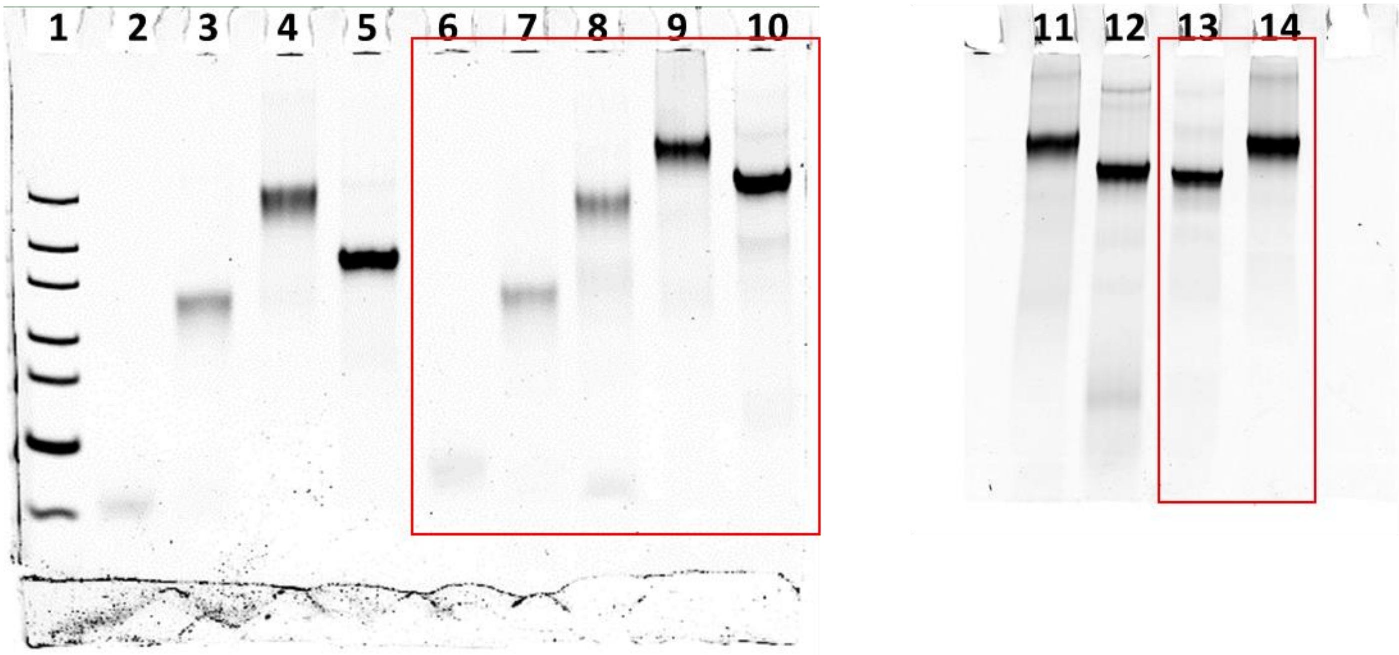

1. ultra-low range DNA ladder
2. Extended 3WJ A-6 PTXs
3. Extended 3WJ A+B-12 PTXs
4. Extended 3WJ A+B+C-18 PTXs (Extended 3WJ-18 PTXs)
5. Extended 3WJ A+B+C (Extended 3WJ)
6. 4WJ A-6 PTXs
7. 4WJ A+B-12 PTXs
8. 4WJ A+B+C-18 PTXs
9. 4WJ A+B+C+D-24 PTXs (4WJ-X-24 PTXs)
10. 4WJ A+B+C+D (4WJ-X)
11. 4WJ A+B+C+D-24 PTXs-EGFR$_{apt}$-ALexa647 (4WJ-X-24 PTXs-EGFR$_{apt}$-Alexa647)
12. 4WJ A+B+C+D-24 PTXs-Alexa647 (4WJ-X-24 PTXs-Alexa647)
13. 4WJ A+B+C+D-24 PTXs (4WJ-X-24 PTXs)
14. 4WJ A+B+C+D-24 PTXs-EGFR$_{apt}$ (4WJ-X-24 PTXs-EGFR$_{apt}$)

Figure 4a

3WJ-10 PTXs

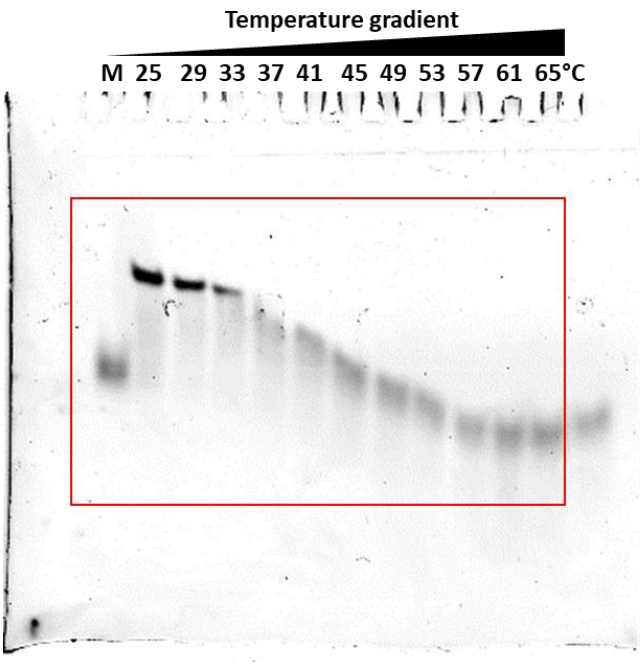

4WJ-X-24 PTXs

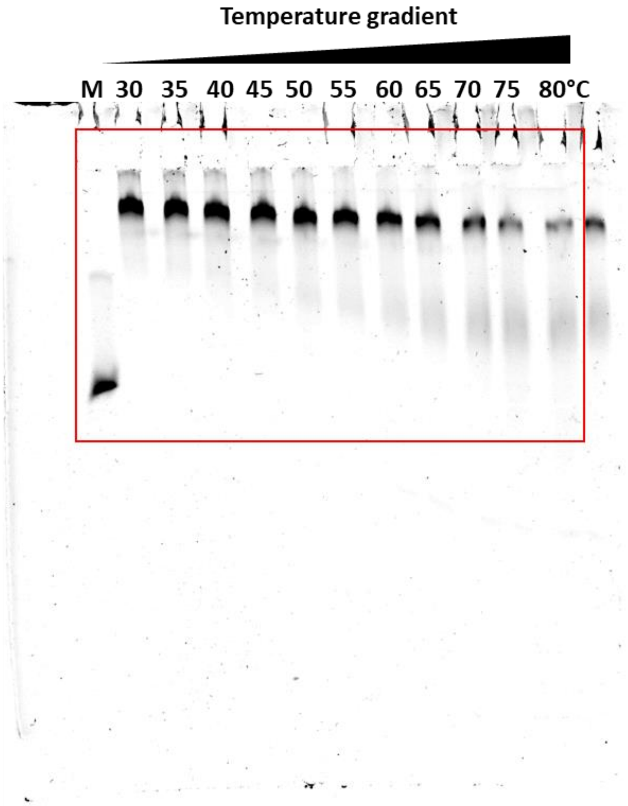

Supplementary Figure 1a

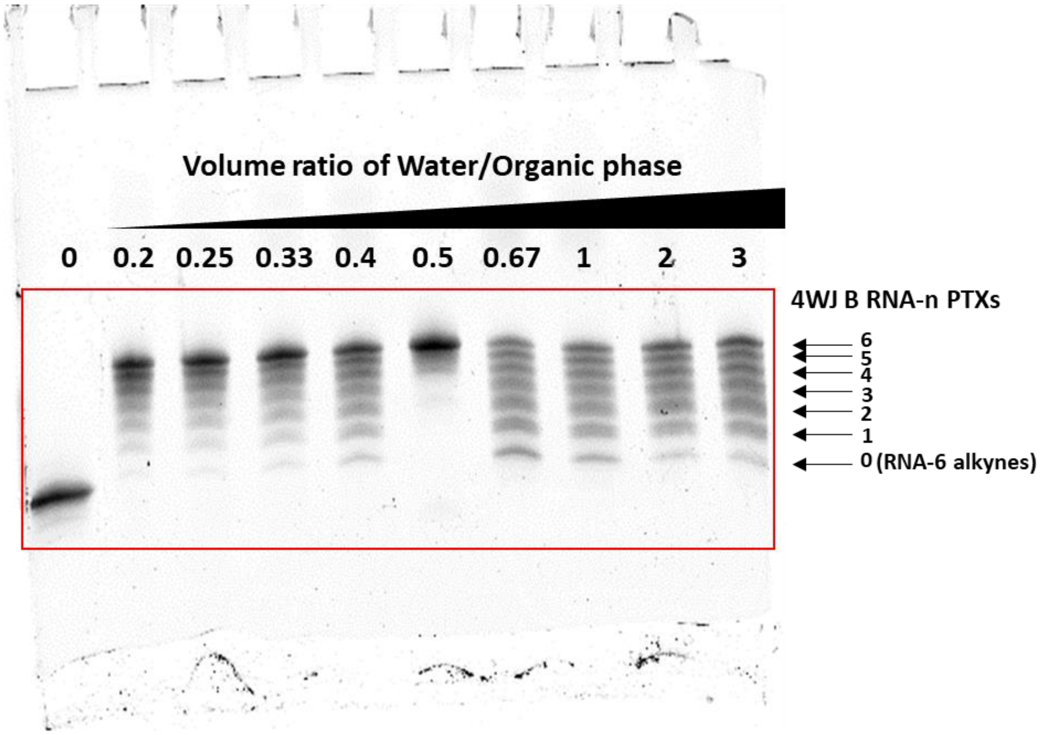

Supplementary Figure 1b

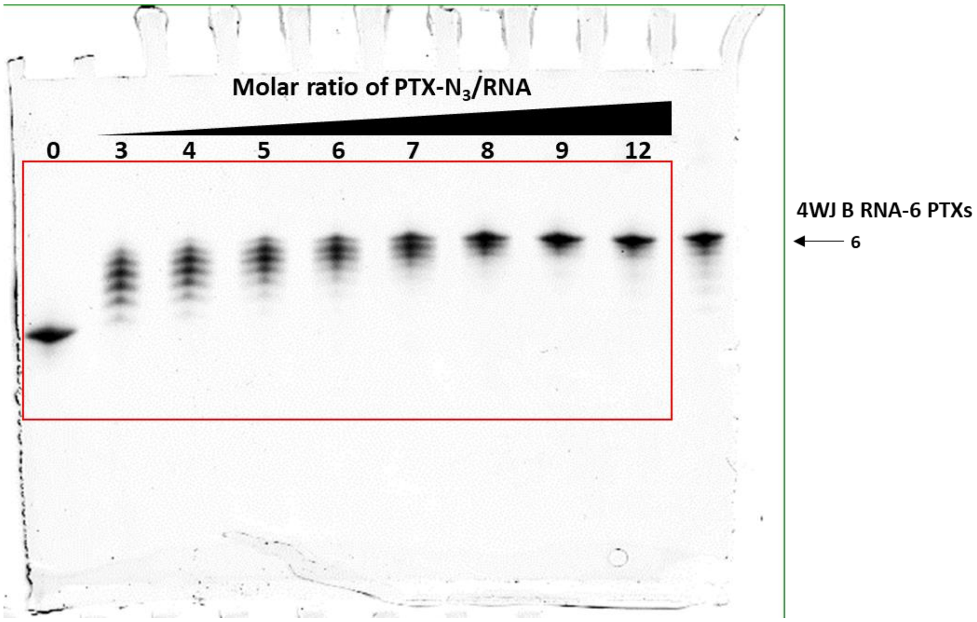

Supplementary Figure 2c

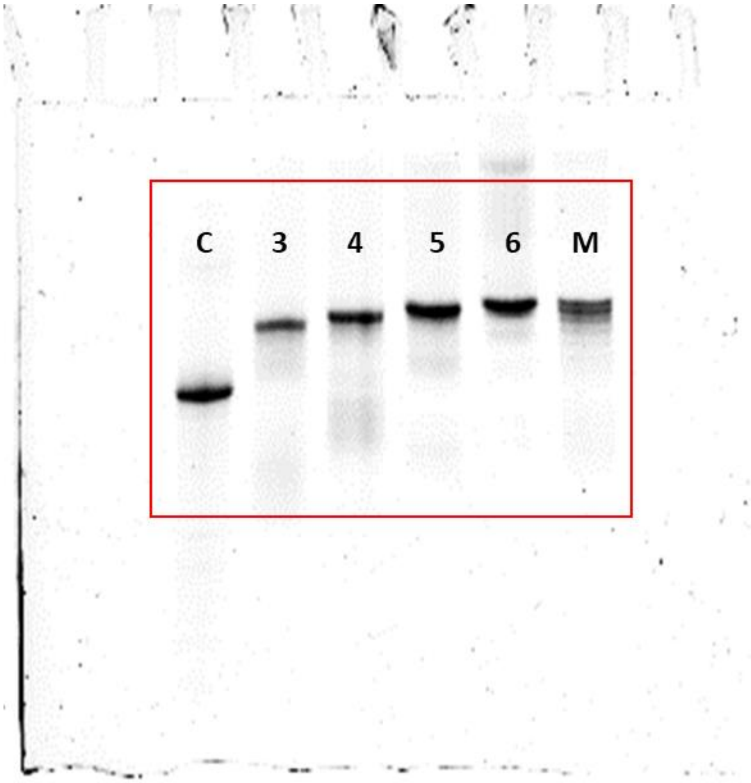

C. RNA-6 alkynes control
3. RNA-3 PTXs
4. RNA-4 PTXs
5. RNA-5 PTXs
6. RNA-6 PTXs
M. RNA-multi-PTXs (before HPLC)

Supplementary Figure 4

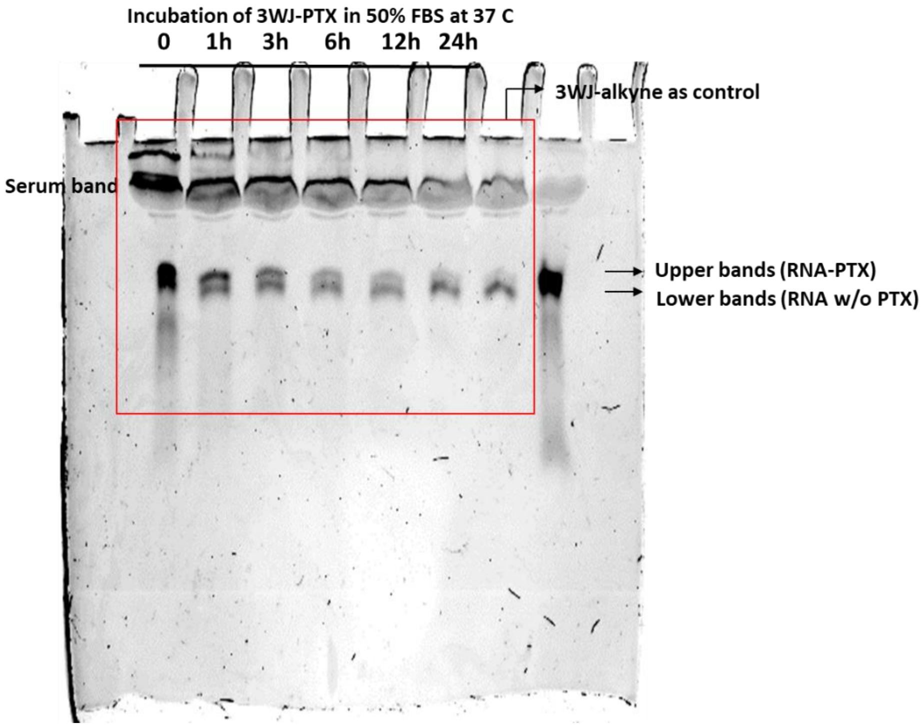

Supplementary Figure 6

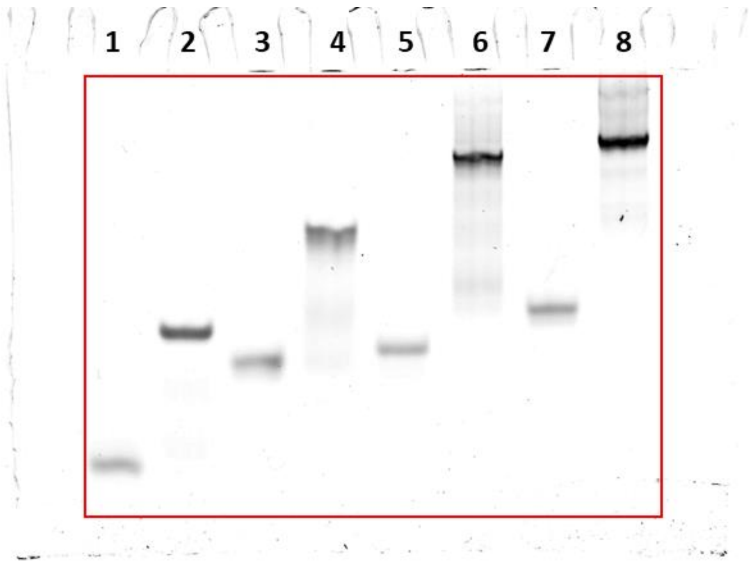

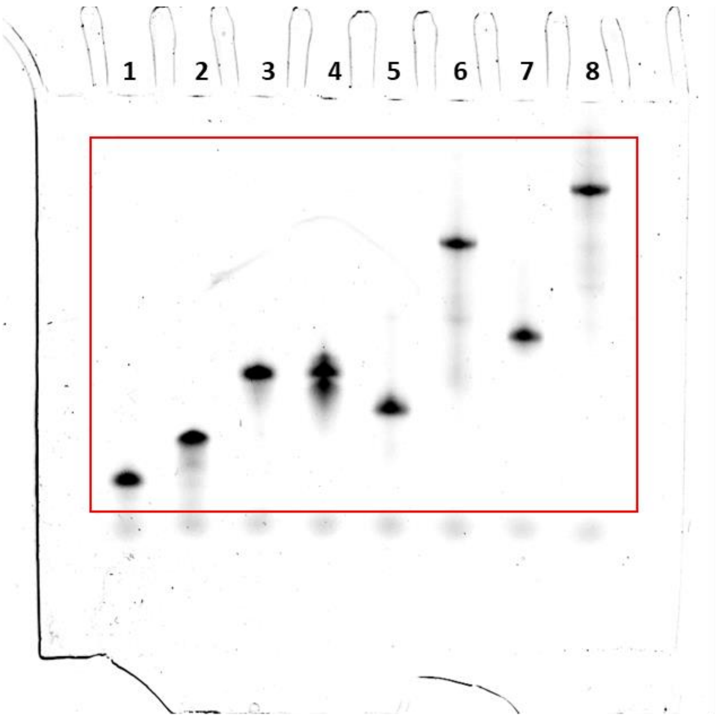

1. 3WJ ssRNA
2. 3WJ
3. 3WJ ssRNA-4 PTXs
4. 3WJ-10 PTXs
5. 4WJ-X-ssrNA
6. 4WJ-X
7. 4WJ-X-ssRNA-6 PTXs
8. 4WJ-X-24 PTXs

Supplementary Figure 7

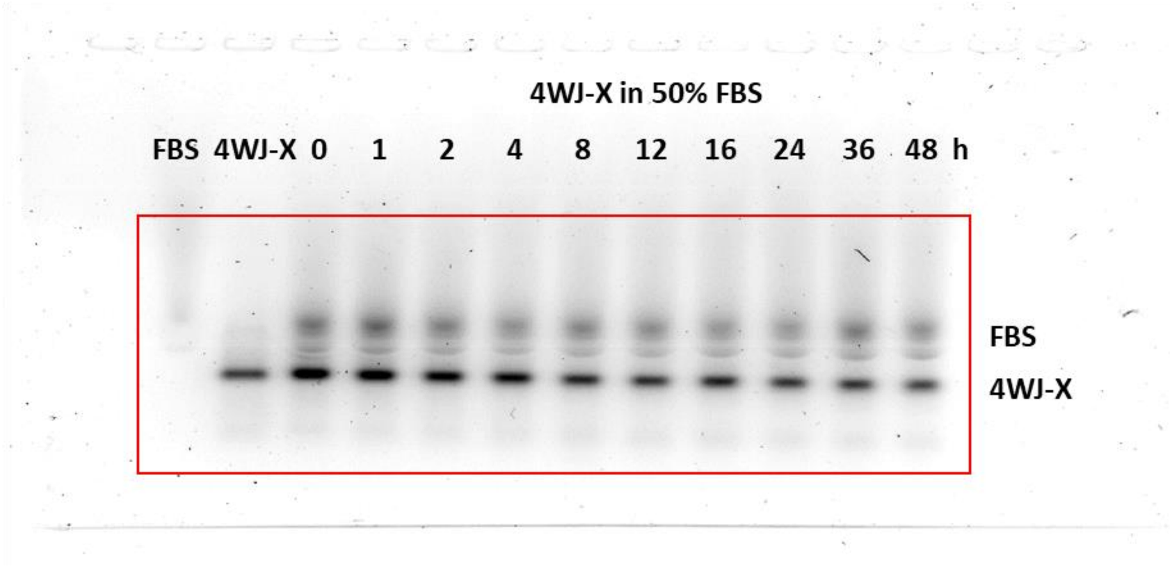

Supplementary Figure 9a

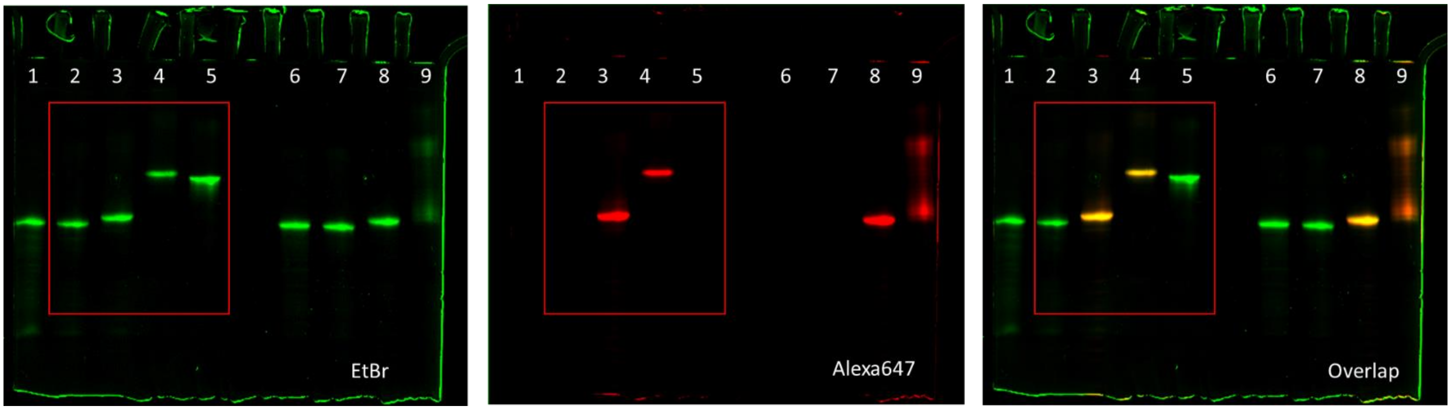

1. 4WJ B RNA-6 alkynes
2. 4WJ B RNA-6 alkynes-NH_2
3. 4WJ B RNA-6 alkynes-Alexa647
4. 4WJ B RNA-6 PTXs-Alexa647
5. 4WJ B RNA-6 PTXs
6. 4WJ B RNA-6 alkynes
7. 4WJ B RNA-6 alkynes-NH_2
8. 4WJ B RNA-6 alkynes-Alexa647
9. 4WJ B RNA-6 PTXs-Alexa647

Supplementary Figure 9b

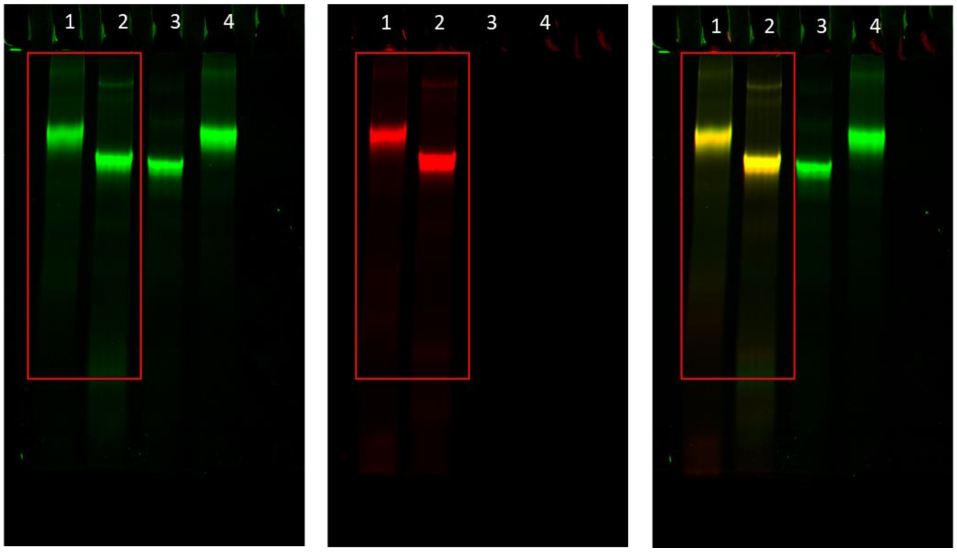

1. 4WJ-X-24 PTXs-$EGFR_{apt}$-Alexa647
2. 4WJ-X-24 PTXs-Alexa647
3. 4WJ-X-24 PTXs
4. 4WJ-X-24 PTXs-$EGFR_{apt}$

Supplementary Figure 13a

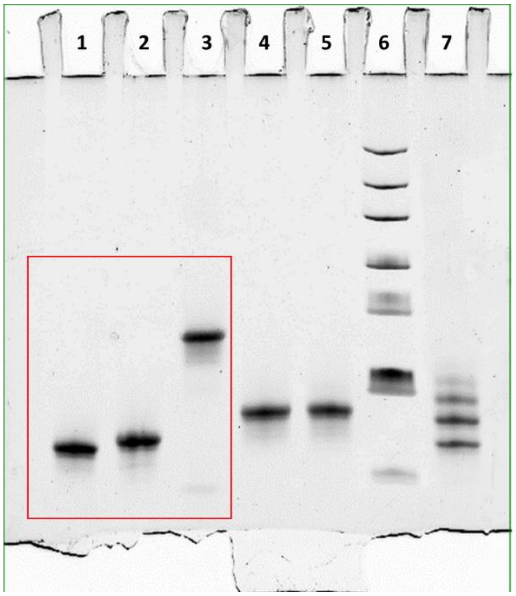

1. 4WJ C-6 alkynes
2. 4WJ C-6 alkynes-5'Folate
3. 4WJ C-6 PTXs-5'Folate
4. 4WJ B-6 alkynes
5. 4WJ B-6 alkynes
6. ultra-low range DNA ladder
7. 4WJ C-multi-PTXs (incomplete conjugation)

Supplementary Figure 13b

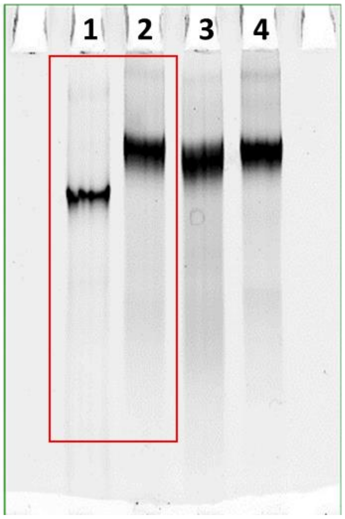

1. 4WJ-X-Folate
2. 4WJ-X-24 PTXs-Folate, 1st batch
3. 4WJ-X-24 PTXs-Folate, 2nd batch
4. 4WJ-X-24 PTXs-Folate, 3rd batch

Supplementary Figure 14a

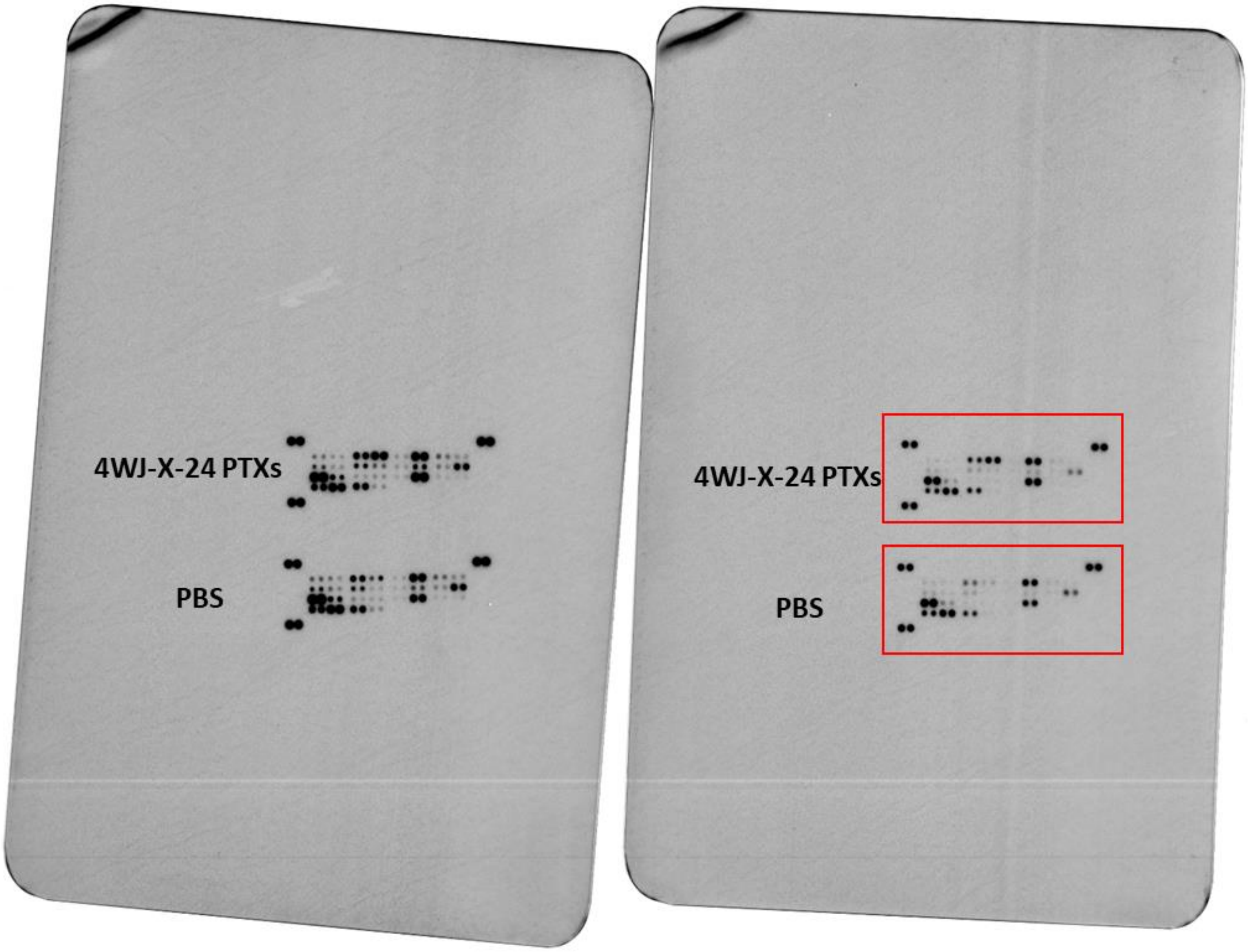

Supplement: Supplementary file 1 — Supplementary Information [file 41467_2020_14780_MOESM1_ESM.pdf]
