# Supplementary material for: Ultra-thermostable RNA nanoparticles for solubilizing and high-yield loading of paclitaxel for breast cancer therapy
Source: Nat Commun. 2020 Feb 20;11:972. doi: 10.1038/s41467-020-14780-5 (PMC7033104; doi:10.1038/s41467-020-14780-5)
Supplement: Supplementary file 2 — Description of Additional Supplementary File [file 41467_2020_14780_MOESM2_ESM.pdf]

File Name: **Supplementary Movie 1**

Description: **3D reconstructed cryo-EM maps of 4WJ-X and 4WJ-24 PTXs nanoparticles.**
