# Supplementary material for: Ultra-thermostable RNA nanoparticles for solubilizing and high-yield loading of paclitaxel for breast cancer therapy
Source: Nat Commun. 2020 Feb 20;11:972. doi: 10.1038/s41467-020-14780-5 (PMC7033104; doi:10.1038/s41467-020-14780-5)
Supplement: Supplementary file 4 — Reporting Summary [file 41467_2020_14780_MOESM4_ESM.pdf]

nature research

Corresponding author(s): Peixuan Guo

Last updated by author(s): Jan 19, 2020

Reporting Summary

Nature Research wishes to improve the reproducibility of the work that we publish. This form provides structure for consistency and transparency in reporting. For further information on Nature Research policies, see Authors & Referees and the Editorial Policy Checklist .

Statistics

For all statistical analyses, confirm that the following items are present in the figure legend, table legend, main text, or Methods section.

n/a	Confirmed	
☐	☒	The exact sample size (*n*) for each experimental group/condition, given as a discrete number and unit of measurement
☐	☒	A statement on whether measurements were taken from distinct samples or whether the same sample was measured repeatedly
☐	☒	The statistical test(s) used AND whether they are one- or two-sided<br>*Only common tests should be described solely by name; describe more complex techniques in the Methods section.*
☒	☐	A description of all covariates tested
☒	☐	A description of any assumptions or corrections, such as tests of normality and adjustment for multiple comparisons
☐	☒	A full description of the statistical parameters including central tendency (e.g. means) or other basic estimates (e.g. regression coefficient) AND variation (e.g. standard deviation) or associated estimates of uncertainty (e.g. confidence intervals)
☐	☒	For null hypothesis testing, the test statistic (e.g. *F*, *t*, *r*) with confidence intervals, effect sizes, degrees of freedom and *P* value noted<br>*Give P values as exact values whenever suitable.*
☒	☐	For Bayesian analysis, information on the choice of priors and Markov chain Monte Carlo settings
☒	☐	For hierarchical and complex designs, identification of the appropriate level for tests and full reporting of outcomes
☒	☐	Estimates of effect sizes (e.g. Cohen's *d*, Pearson's *r*), indicating how they were calculated

Our web collection on statistics for biologists contains articles on many of the points above.

Software and code

Policy information about availability of computer code

Data collection	3D RNA Model - Swiss-PdbViewer V4.1 and PyMOL V2.0.0<br>Gel Images - ImageQuant TL V7.0<br>Dynamic Light Scattering (Size and Zeta Potential) - Zetasizer V7.11<br>HPLC - OpenLAB CDS V1.12<br>Cryo-EM - EPU V1<br>Real-time PCR - LightCycler® 480 V1.5<br>Flow Cytometry - FACSCalibur<br>Confocal Microscopy - Fluoview FV31S-SW<br>Cell Viability - Gen5 V1.09<br>Biodistribution - IVIS system (XMRS)

Data analysis	Gel Images - ImageQuant TL V7.0 & ImageJ V1.51<br>Dynamic Light Scattering (Size and Zeta Potential) - Zetasizer V7.11<br>HPLC - OpenLAB CDS V1.12<br>Cryo-EM - Motioncor2 V1.0.5, CTFFIND V4.1, EMAN2 V2.2, RELION V2.1, Chimera V1.13.1, CryoSPARC V0.65<br>Real-time PCR - LightCycler® 480 V1.5<br>Flow Cytometry - FlowJo V7.6.2<br>Confocal Microscopy - Fluoview FV31S-SW<br>Quantitative Analysis - Origin V9.0<br>Biodistribution - IVIS system (XMRS) and Living Image V4.5.2<br>Tumor Volume and Weight Curves - GraphPad Prism V8.3.0.538<br>Statistics - GraphPad Prism V8.3.0.538 and Origin V9.0

For manuscripts utilizing custom algorithms or software that are central to the research but not yet described in published literature, software must be made available to editors/reviewers. We strongly encourage code deposition in a community repository (e.g. GitHub). See the Nature Research guidelines for submitting code & software for further information.

Data

Policy information about availability of data

All manuscripts must include a data availability statement. This statement should provide the following information, where applicable:

- Accession codes, unique identifiers, or web links for publicly available datasets
- A list of figures that have associated raw data
- A description of any restrictions on data availability

The source data underlying Figures 1-7 and Supplementary Figure 1-14 are provided in the source data file. Cryo-EM structures have been deposited to the Electron Microscopy Data Bank under accession codes EMD-20699 for 4WJ-X nanoparticles and EMD-20697 for 4WJ-X-24 PTXs nanoparticles, respectively. Other relevant data that support the findings of this study are available from the corresponding author upon reasonable request.

Field-specific reporting

Please select the one below that is the best fit for your research. If you are not sure, read the appropriate sections before making your selection.

☒ Life sciences ☐ Behavioural & social sciences ☐ Ecological, evolutionary & environmental sciences

For a reference copy of the document with all sections, see nature.com/documents/nr-reporting-summary-flat.pdf

Life sciences study design

All studies must disclose on these points even when the disclosure is negative.

Sample size	Due to the high reproducibility and consistency between cell cultures, a sample size of n=3 would allow for adequate analysis to reach meaningful conclusions of the data for in vitro studies. However, due to higher variance for in vivo studies, a higher sample size (n=5) is used to compensate for this natural variance. In previous studies using related experiments, these sample sizes has been determined to be sufficient to ensure reproducibility (Pi, F. et al., Nanoparticle orientation to control RNA loading and ligand display on extracellular vesicles for cancer regression. Nat Nanotechnol. 13, 82-89 (2018).)
Data exclusions	No data was excluded from studies.
Replication	Each experiment was repeated independently for at least three times for each sample tested, unless otherwise indicated. All replication of experiments was successful.
Randomization	Samples and animals were randomized into groups throughout the experiments.
Blinding	All in vivo data collected was quantifiable and tested by statistics. Thus, no blinding was used and would not change bias in the study.

Reporting for specific materials, systems and methods

We require information from authors about some types of materials, experimental systems and methods used in many studies. Here, indicate whether each material, system or method listed is relevant to your study. If you are not sure if a list item applies to your research, read the appropriate section before selecting a response.

Materials & experimental systems

n/a	Involved in the study	
☐	☒	Antibodies
☐	☒	Eukaryotic cell lines
☒	☐	Palaeontology
☐	☒	Animals and other organisms
☒	☐	Human research participants
☒	☐	Clinical data

Methods

n/a	Involved in the study	
☒	☐	ChIP-seq
☐	☒	Flow cytometry
☒	☐	MRI-based neuroimaging

Antibodies

Antibodies used

Antibodies used for ELISA include Mouse TNF-α Capture Antibody (200X) and Detection Antibody (200X) from ELISA MAX™ Deluxe Set Mouse TNF-α kit (BioLegend, Cat.# 430904), Mouse IL-6 Capture Antibody (200X) and Detection Antibody (200X) from ELISA MAX™ Deluxe Set Mouse IL-6 kit (BioLegend, Cat.# 431304), Mouse IFN-γ Capture Antibody (200X) and Detection Antibody (200X) from ELISA MAX™ Deluxe Set Mouse IFN-γ kit (BioLegend, Cat.# 430804), and Mouse IFN-α Detection Antibody (200X) from Mouse IFN-alpha ELISA Kit (R&D Systems, Cat.# 42120-2).

Validation

Antibodies in commercially available ELISA kits were validated by the manufacturer. Validation statement is provided on the manufacturer's website.

Eukaryotic cell lines

Policy information about cell lines

Cell line source(s)

MDA-MB-231 cells and KB cells were obtained from American Type Culture Collection (ATCC).

Authentication

Cell lines purchased from ATCC were authenticated by Short Tandem Repeat (STR) Profiling Service.

Mycoplasma contamination

Cell lines were not tested for mycoplasma contamination.

Commonly misidentified lines
(See ICLAC register)

KB cell line, listed as a misidentified cell line, was used in this work. It is known to be a subline of the ubiquitous KERATIN-forming tumor cell line HeLa which is sensitive to paclitaxel. Thus, it serves as an ideal model in this study.

Animals and other organisms

Policy information about studies involving animals; ARRIVE guidelines recommended for reporting animal research

Laboratory animals

4-8 week-old NCR nude female mice were purchased from Taconic BiosciencesFarm.

Wild animals

No wild animals were used.

Field-collected samples

No field-collected samples were used.

Ethics oversight

All animal procedures were housed and performed in accordance with the Subcommittee on Research Animal Care of The Ohio State University guidelines approved by the Institutional Review Board.

Note that full information on the approval of the study protocol must also be provided in the manuscript.

Flow Cytometry

Plots

Confirm that:

- ☒ The axis labels state the marker and fluorochrome used (e.g. CD4-FITC).
- ☒ The axis scales are clearly visible. Include numbers along axes only for bottom left plot of group (a 'group' is an analysis of identical markers).
- ☒ All plots are contour plots with outliers or pseudocolor plots.
- ☒ A numerical value for number of cells or percentage (with statistics) is provided.

Methodology

Sample preparation

Cells were seeded on 24-well plates overnight and then treated with different samples. After 48 h incubation, cells were trypsinized to single cell suspension. After two times PBS wash, the cells were re-suspended in 100 µL 1x Annexin V-FITC binding buffer. Then 5 µL AnnexinV-FITC and 5 µL propidium iodide (PI) were added into each sample and incubated at room temperature for 25 min. The samples were finally added to a flow tube which contained 200 µL 1x binding buffer for FACS

	analysis within 1 h.
Instrument	Samples were analyzed by FACSCalibur with four lasers.
Software	Flow cytometry data was analyzed using FlowJo V7.6.2.
Cell population abundance	During sample measurements, the initial gate was used to ensure a cell count of 10,000.
Gating strategy	Initial cell populations were gated for a population using FSC and SSC plot of unstained sample. The gate (P1) was set to remove cell debris (small FSC v SSC) and large clumps or aggregate of cells (large FSC or SSC) and used across all samples. The gate of apoptosis population follows instruction of BD Pharmingen FITC Annexin V Apoptosis Detection Kit I. The populations are classified into: early apoptotic cells (Q3: PI negative, FITC Annexin V positive), late apoptosis or already dead cells (Q2: FITC Annexin V and PI positive), live cells (Q4: FITC Annexin V and PI negative). The position of gate is based on single stained and double stained cells only population, which should have minimized PI & FITC Annexin V positive population.

☒ Tick this box to confirm that a figure exemplifying the gating strategy is provided in the Supplementary Information.
